# Is there an inflammatory stimulus to human term labour?

**DOI:** 10.1371/journal.pone.0256545

**Published:** 2021-08-31

**Authors:** Natasha Singh, Bronwen Herbert, Garvin Sooranna, Nishel M. Shah, Ananya Das, Suren R. Sooranna, Mark R. Johnson

**Affiliations:** 1 Chelsea and Westminster Hospital, London, United Kingdom; 2 Department of Metabolism, Digestion and Reproduction, Imperial College London, London, United Kingdom; BC Children’s Hospital, CANADA

## Abstract

Inflammation is thought to play a pivotal role in the onset of term and some forms of preterm labour. Although, we recently found that myometrial inflammation is a consequence rather than a cause of term labour, there are several other reproductive tissues, including amnion, choriodecidua parietalis and decidua basalis, where the inflammatory stimulus to labour may occur. To investigate this, we have obtained amnion, choriodecidual parietalis and decidua basalis samples from women at various stages of pregnancy and spontaneous labour. The inflammatory cytokine profile in each tissue was determine by Bio-Plex Pro® cytokine multiplex assays and quantitative RT-PCR. Active motif assay was used to study transcription activation in the choriodecidua parietalis. Quantitative RT-PCR was use to study the pro-labour genes (*PGHS-2*, *PGDH*, *OTR and CX43*) in all of the tissues at the onset of labour and *oxytocin (OT)* mRNA expression in the choriodecidual parietalis and decidua basalis. Statistical significance was ascribed to a P value <0.05. In the amnion and choriodecidua parietalis, the mRNA levels of various cytokines decreased from preterm no labour to term no labour samples, but the protein levels were unchanged. The choriodecidua parietalis showed increase in the protein levels of IL-1β and IL-6 in the term early labour samples. In the amnion and decidua basalis, the protein levels of several cytokines rose in term established labour. The multiples of the median derived from the 19-plex cytokine assay were greater in term early labour and term established labour samples from the choriodecidua parietalis, but only in term established labour for myometrium. These data suggest that the inflammatory stimulus to labour may begin in the choriodecidua parietalis, but the absence of any change in prolabour factor mRNA levels suggests that the cytokines may act on the myometrium where we observed changes in transcription factor activation and increases in prolabour gene expression in earlier studies.

## Introduction

Term human labour has been suggested to be an inflammatory event induced by an interaction between immunological, humoral and mechanical factors [1]. Indeed, the seminal papers of Norman et al. supported this view showing a marked myometrial inflammatory infiltration consisting predominantly of neutrophils and macrophages [2] and latterly providing a convincing mechanism through which monocytes might enhance myometrial contractility [3]. Earlier myometrial inflammation was found in samples obtained at the time of emergency Caesarean Section by Azziz *et al*., who suggested that this might reflect an underlying infective aetiology, but the infective component was later discounted [4–7]. However, since most of the samples in all of the studies mentioned above were obtained after labour was well established, the question remained whether inflammation initiated or was simply a response to labour. Recently, we attempted to answer this question by studying myometrial samples obtained in early labour and found that there was no evidence of inflammation in these samples, demonstrating that myometrial inflammation was most likely to be a consequence rather than a cause of the onset of labour [8]. However, inflammation has been described not only in the myometrium but also in the fetal membranes and decidua [4]. Progressive stretch of the fetal membranes may drive these changes [9], alternatively, recently an altered immune response has been reported to occur prior to the onset of labour specifically in the zone of altered morphology [10]. Further, decidual inflammatory gene networks have been implicated in the onset of labour [11], consistent with the description of a decidual macrophage infiltration prior to the onset of labour [12]. This has been related to changes in decidual chemokine expression [13] and may be due to inflammation in the amnion or choriodecidua that may drive the expression of prostaglandin synthetic enzymes [14] or oxytocin [15] and the onset of labour.

Our earlier data suggested that myometrial inflammation does not precede the onset of labour, but it is still possible that inflammation in other reproductive tissues occurs prior to the onset of labour and may have a role in its onset. For example, inflammation may increase prostaglandin levels (by increasing their synthesis in the amnion or by reducing their metabolism in the chorion or by increasing local oxytocin release from the decidua or the responsiveness of the myometrium to oxytocin by increasing the level of the oxytocin receptor in the myometrium [16–19]. The expression of these prolabour genes is controlled by a group of inflammation-related transcription factors including NFkB, AP-1, C/EBP [20] and changes in their activity with labour have been reported in human and animal studies [21, 22].

In this study, we have extended our original hypothesis that myometrial inflammation drives the onset of labour, by testing the hypothesis that inflammation in other reproductive tissues may precede and have a role in the onset of labour. We have achieved this by comparing the levels of inflammatory cytokines in the amnion, choriodecidua parietalis and decidua basalis from women at various stages of pregnancy and spontaneous labour. In addition, we examined the expression of a group of recognised prolabour genes *(PGHS-2*, *PGDH*, *OTR and CX43)* in the same reproductive tissues to allow us to relate any changes in the inflammatory signal to increased prolabour gene expression.

## Material and methods

### Ethical approval

The study has been approved by the South West London Research Ethics Committee, 10/H0801/45. Written consent was obtained from all participants.

### Tissue collection

All procedures involving human tissues were conducted in compliance with the London-Chelsea Ethics committee. Informed consent was obtained from all women prior to any tissue collection. Labour was defined as the presence of regular uterine contractions every 3–4 minutes. To study the inflammatory changes during pregnancy, at the onset of labour and after labour occurred it was important to further define labour into early labour and established labour. Term early labour was defined as a cervical dilatation of 3 cm or less and term established labour as a cervical dilatation of more than 3 cm. In all of the labouring women, the onset of labour was spontaneous and the progress of labour was normal. Women in whom oxytocin was used or who had prolonged rupture of membranes or chorioamnionitis were excluded.

Samples were obtained from 4 groups of women at preterm no labour (PTNL), term no labour (TNL), term early labour (TeL) and term established labour (TestL). The samples were taken from the same cohort of women in which we have previously studied and report the inflammatory changes in the myometrium [8]. The indications for the Caesarean section were for previous Caesarean section, breech presentation or fetal distress. The demographics of the women are summarised in S1 and S2 Tables. From each woman amnion, choriodecidual parietalis and decidua basalis samples were collected at the time of Caesarean section. The decidua basalis overlaying the maternal side of the placenta was removed and 1 cm^3^ pieces of placental tissue cut from various sites across the placenta midway between the cord and periphery membranes were detached from the placenta. The choriodecidua parietalis and amnion were separated from each other by blunt dissection. All the samples were collected and frozen immediately at -80°C.

### Bio-Plex Pro® cytokine multiplex assays

Protein lysates were prepared from the frozen amnion tissues from women (mean gestational age ± SD in each case), at preterm no labour (PTNL; 33.8± 1.7 weeks, n = 12), term no labour (TNL; 39.3± 0.9 weeks, n = 13), term early labour (TeL; 38.6 ± 1.2 weeks, n = 15) and term established labour (TestL; 39.6 ± 1.2 n = 11). Similarly, protein lysate was also prepared from frozen choriodecidua parietalis and decidua basalis samples from women at preterm no labour (PTNL; 33.8± 1.7 weeks, n = 17), term no labour (TNL; 39.3± 0.8 weeks, n = 19), term early labour (TeL; 38.4 ± 1.3 weeks, n = 21) and term established labour (TestL; 39.5 ± 1.0, n = 15). The sample size used differ due to tissue availability.

The lysates were prepared using the Bio-Plex Pro^TM^ cell signalling reagent kit (BioRad, Hemel Hempstead, UK) and the Precellys®24 Dual bead homogeniser system, according to the manufacturer’s instructions. Lysate concentrations were quantified by DC^TM^ Protein Assay (BioRad, Hemel Hempstead, UK). 500μg of protein lysate was added per well to a Bio-Plex® 19-plex^TM^ (analytes listed in S3 Table) and due to buffer incompatibility, onto an additional separate Bio-Plex® *CCL5* single-plex assay for each tissue type. One of the risks is that of intraplate variation among the different tissues, but we attempted to reduce this by making sure all of the samples from the same tissue were on the same plate. The samples were done in singlicate due to the number of samples available. Appropriate standards and controls were provided with the assays and both were completed in accordance with the manufacturer’s instructions. The cytokine concentrations in the amnion protein lysate were calculated using an 8-point calibration curve for each individual analyte produced from manufacturer-supplied set standards of known concentration and presented as concentrations (pg/ml).

### Total RNA extraction, cDNA synthesis and rt-PCR of human samples

A subset of the human tissue samples was used to study the mRNA expression of the pro-inflammatory (*IL-1*β, *TNFα*, *IL6*), chemotactic (*CCL2*, *CCL5*, *CXCL1*, *CXCL2*, *IL8*) and anti-inflammatory (*IL4*, *IL10*) chemokines and prolabour genes (*PGHS2*, *OTR*, *oxytocin*, *Cx43*) from women PTNL, TNL, TeL. Oxytocin mRNA was only studied in the choriodecidual parietalis and decidua basalis as these tissues have been previously shown to be associated changes in the regulation of *OT* synthesis [23] and increased *OT* expression at the onset of labour [15, 24].

Amnion, choriodecidual parietalis and decidua basalis samples were homogenised using the Precellys®24 Dual system and CK Mix tubes (Bertin Instruments, France) in RNA Stat (AMS Biotechnology, UK) according to the manufacturer’s instructions. After chloroform addition and centrifugation, RNA extraction from the supernatant was completed using an RNeasy mini kit (Qiagen Ltd, UK), as per the manufacturer’s instructions. RNA quality and quantity were assessed using a Nanodrop (Thermo Scientific, USA). After RNA quantification, 1.0ug was reverse transcribed with oligo dT random primers using the MuLV reverse transcriptase system (Applied Biosystems Ltd., Warrington, Cheshire, UK), according to the manufacturer’s protocols.

Primer sets for genes listed in S4 Table were designed and obtained from Invitrogen Ltd. (Paisley, UK). Assays were validated for all primer sets by confirming that single amplicons of appropriate size and sequence were generated according to predictions. Quantitative PCR was performed in the presence of SYBR Green (Applied Biosystems Ltd.), and amplicon yield was monitored during cycling in a RotorGene Sequence Detector (Corbett Research Ltd., Mortlake, Sydney, Australia) that continually measures fluorescence caused by the binding of the dye to double-stranded DNA. Pre-PCR cycle was 10 minutes at 95°C followed by up to 45 cycles of 95°C for 20 seconds, 58–60°C for 20 seconds and 72°C for 20 seconds followed by an extension at 72°C for 15 seconds. The final procedure involves a melt over the temperature range of 72–99°C rising by 1° steps with a wait for 15 seconds on the first step followed by a wait of 5 seconds for each subsequent step. The cycle in which fluorescence reached a pre-set threshold (cycle threshold) was used or quantitative analyses. The cycle threshold in each assay was set at a level where the exponential increase in amplicon abundance was approximately parallel between all samples.

For each tissue the most stable housekeeping gene was determined by geNORM [25]. For the placental, choriodecidual and amnion samples each gene was expressed relative to the amount of constitutively expressed normalisation factor, which was the average most stable of the housekeeping genes for each tissue (Placenta: *GAPDH*, *CYC*, *BM2;* Choriodecidua: *GAPDH*, *CYC*, *B-actin;* amnion: *GAPDH)*.

### Active Motif assay

Relative levels of *Phospho-cJun*, *Phospho-NFKB p65* and *Phospho-CREB* in the choriodecidual samples were measured using TransAMTM *NFKB* and TransAMTM *AP1* transcription factor DNA-protein binding assays (Active Motif, Carlsbad CA, USA) from women (mean gestational age ± SD in each case), at preterm no labour (PTNL; 33.2 ± 2.5 weeks, n = 18), term no labour (TNL; 39.5 ± 0.8 weeks, n = 19), early labour (38.3 ± 1.1 weeks, n = 18) and term established labour (39.5 ± 1.0, n = 15).

Whole cell lysates were prepared using a Precellys®24 bead homogeniser (Stretton Scientific Ltd, UK), with the Active Motif Nuclear Extraction Kit, in accordance with the manufacturer’s instructions for preparations from frozen tissues. Protein concentrations were quantified using a DC Protein Assay (Bio-Rad, Hemel Hempstead, UK). 100μg and 200μg of protein lysate was added per sample well for the TransAMTM *AP1* and TransAMTM *NFKB* respectively, diluted in the appropriate individual transcription factor assay lysis buffer. Assays were completed according to the manufacturer’s instructions.

### Statistical analysis

All data were initially tested for normality using a Kolmogorov–Smirnoff test. Normally distributed data were analysed using a Student’s t-test for two groups and an ANOVA followed by a Dunnett’s or Bonferroni’s post-hoc test for three groups or more. Data that were not normally distributed were analysed using a Wilcoxon matched pair test for paired data and when comparing three groups or more a Friedman’s test, with a Dunn’s multiple comparisons post-hoc test. P <0.05 was considered statistically significant.

## Results

### Demographics of the women

All of the women had a caesarean birth and none of them received oxytocin during their labour. There was no statistical difference among the women for BMI and maternal age. For the term samples there was no statistical difference in the gestational age. The demographics of the women are summarised in S1 and S2 Tables.

### Amnion cytokine profile during pregnancy and in labour

#### Pregnancy

**Fig 1:**
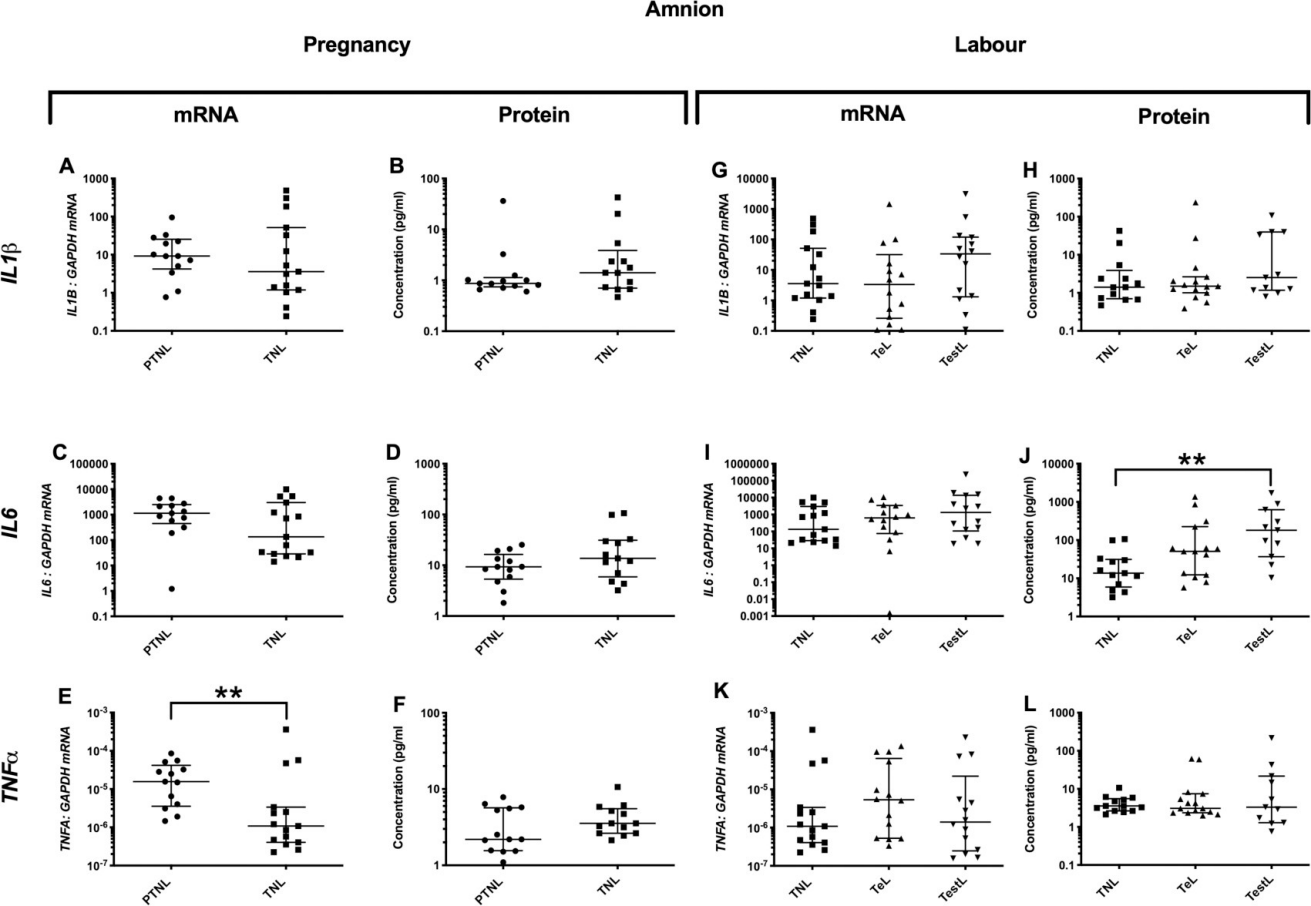
Pro-inflammatory cytokine changes in the amnion. Amnion tissue levels of cytokines from four groups of women (mean gestational age ± SD in each case), PTNL (33.8±1.7 weeks, n = 12), TNL (39.3 ±0.9 weeks, n = 13), TeL (cervical dilatation < 3cm, 38.6±1.2 weeks, n = 15) and TestL (cervical dilatation >3cm, 39.6±1.2, n = 11) were compared. Tissue levels of the cytokines were quantified using Bio-plex human cytokine 19-plex-array kit (Bio-Rad) and a separate assay for *CCL5* as describe in *Materials and Methods*. A subset of amnion samples, PTNL (33.3±1.9 weeks, n = 13), TNL (39.0 ±1.0 weeks, n = 15), TeL 38.5±1.3 weeks, n = 14) and TestL (39.4±1.1, n = 14) were used to compare the mRNA expression using rtPCR of the *IL-1*β, *IL6 and TNFα*. Normally distributed data were analysed using a Student’s t-test for two groups and an ANOVA followed by a Dunnett’s or Bonferroni’s post-hoc test for three groups or more. Data that were not normally distributed were analysed using a Mann Whitney test for 2 groups and when comparing three groups or more a Friedman’s test, with a Dunn’s multiple comparisons post-hoc test. The data are shown as median with interquartile range. The p values are demonstrated by * is p<0.05 and ** is p<0.01.

**Fig 2:**
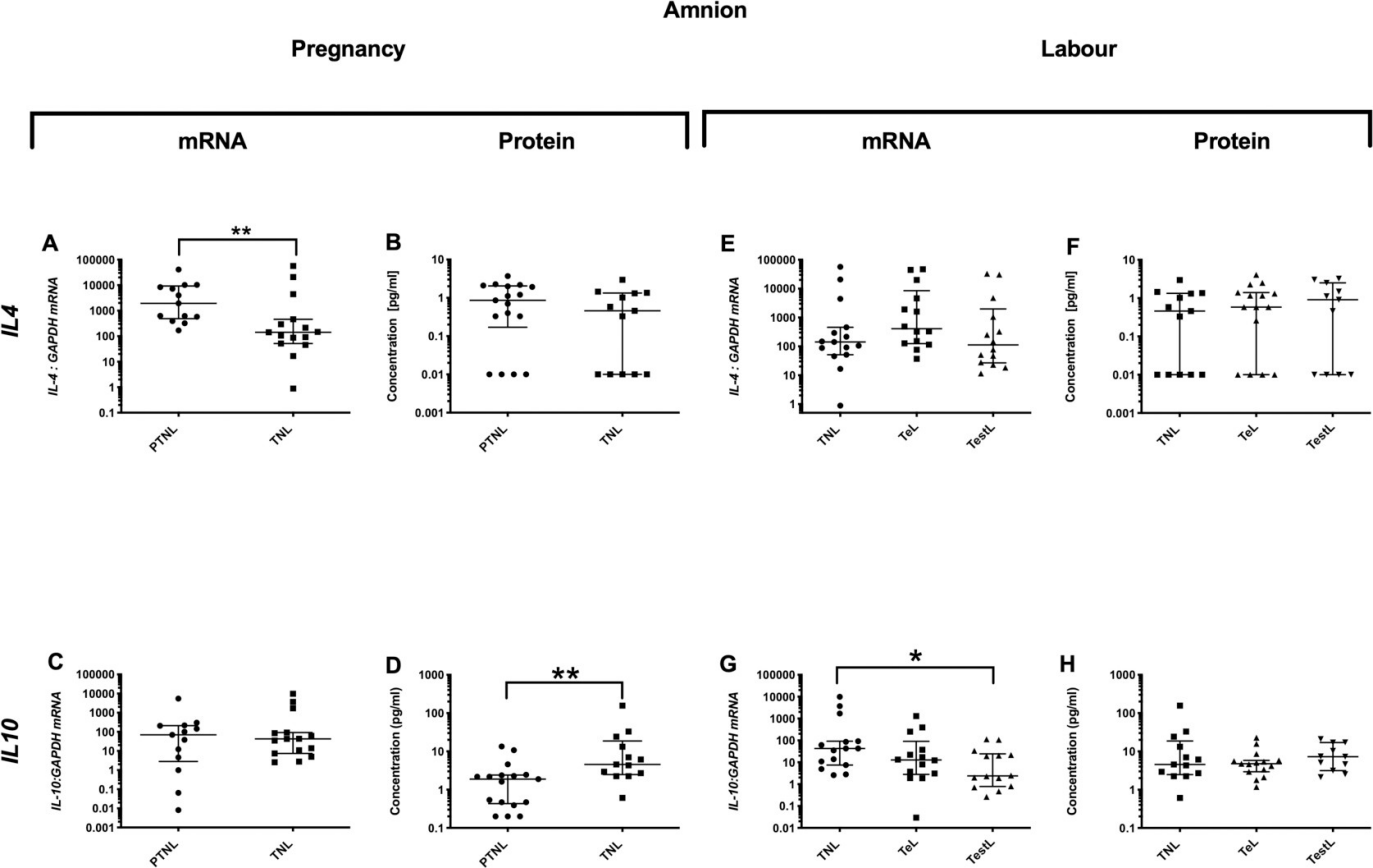
Anti-inflammatory cytokines changes in the amnion. Amnion tissue levels of cytokines from four groups of women (mean gestational age ± SD in each case), PTNL (33.8±1.7 weeks, n = 12), TNL (39.3 ±0.9 weeks, n = 13), TeL (cervical dilatation < 3cm, 38.6±1.2 weeks, n = 15) and TestL (cervical dilatation >3cm, 39.6±1.2, n = 11) were compared. Tissue levels of the cytokines were quantified using Bio-plex human cytokine 19-plex-array kit (Bio-rad) as describe in *Materials and Methods*. A subset of amnion samples, PTNL (33.3±1.9 weeks, n = 13), TNL (39.0 ±1.0 weeks, n = 15), TeL 38.5±1.3 weeks, n = 14) and TestL (39.4±1.1, n = 14) were used to compare the mRNA expression using rtPCR of *IL4 and IL10*. Normally distributed data were analysed using a Student’s t-test and for data that were not normally distributed were analysed using a Mann Whitney test. The data are shown as median with interquartile range. The p values are demonstrated by * is p<0.05 and ** is p<0.01.

**Fig 3:**
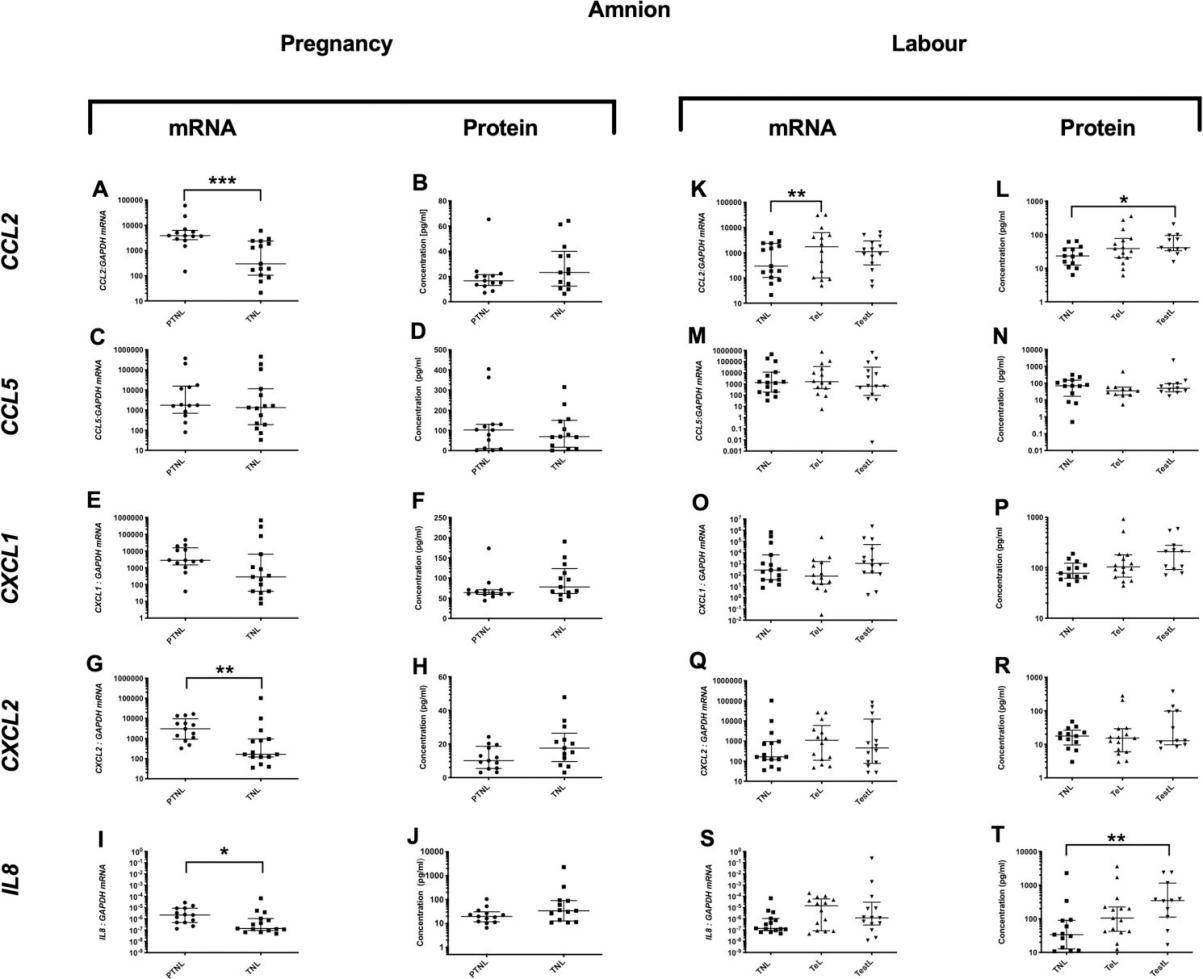
Chemotactic cytokine changes in the amnion. Amnion tissue levels of cytokines from four groups of women (mean gestational age ± SD in each case), PTNL (33.8±1.7 weeks, n = 12), TNL (39.3 ±0.9 weeks, n = 13), TeL (cervical dilatation < 3cm, 38.6±1.2 weeks, n = 15) and TestL (cervical dilatation >3cm, 39.6±1.2, n = 11) were compared. Tissue levels of the cytokines were quantified using Bio-plex human cytokine 19-plex-array kit (Bio-rad) and a separate assay for *CCL5* as describe in *Materials and Methods*. A subset of amnion samples, PTNL (33.3±1.9 weeks, n = 13), TNL (39.0 ±1.0 weeks, n = 15), TeL 38.5±1.3 weeks, n = 14) and TestL (39.4±1.1, n = 14) were used to compare the mRNA expression using rtPCR *CCL2*, *CCL5*, *CXCL1 and CXCL2*, *IL-8*. Normally distributed data were analysed using a Student’s t-test for two groups and an ANOVA followed by a Dunnett’s or Bonferroni’s post-hoc test for three groups or more. Data that were not normally distributed were analysed using a Mann Whitney test for 2 groups and when comparing three groups or more a Friedman’s test, with a Dunn’s multiple comparisons post-hoc test. The data are shown as median with interquartile range. The p values are demonstrated by * is p<0.05 and ** is p<0.01.

*Pro-inflammatory changes*. As the pregnancy advanced the mRNA expression of the pro-inflammatory *TNFα* decreased in the amnion (p<0.01, Fig 1E), but this was not observed at the protein level (Fig 1F). *Anti-inflammatory changes*: *IL4* mRNA (p<0.01, Fig 2A), but not protein, expression was lower (Fig 2B). *IL10* protein concentrations were higher in TNL compared to PTNL samples (Fig 2C and 2D). *Chemotactic changes*: There was a decrease in the chemokine mRNA expression (*CCL2*, *CXCL2* and *IL8*, p<0.001, 0.01 and 0.05 respectively, Fig 3A–3I) from PTNL to TNL samples, but this was not seen at the protein level.

#### Labour

*Pro-inflammatory changes*. *IL-1*β and *TNFα* mRNA or protein levels were unchanged (Fig 1G, 1H, 1K and 1L), but *IL6* protein levels were higher in TestL compared to TNL samples (p<0.01; Fig 1I and 1J). *Anti-inflammatory changes*: There was a lower mRNA expression of *IL10* in TestL compared to the TNL samples (p<0.05), but this decline was not observed in the protein concentration (Fig 2G and 2H). *Chemotactic changes*: The onset of labour was associated with an increase in the mRNA expression of *CCL2* (p<0.01, Fig 3K), but this was only translated into an increase in *CCL2* protein only after labour was established (p<0.05, Fig 3L). Similarly, the protein concentration of *IL8* was higher in TestL vs. TNL samples only (p<0.01, Fig 3T).

#### Other cytokine changes

*In pregnancy*, there was a higher protein concentration of *CCL1* in TNL compared to PTNL (p<0.05, S5 Table); *In labour*, no changes.

### Decidua basalis cytokine profile during pregnancy and in labour

#### Pregnancy

**Fig 4:**
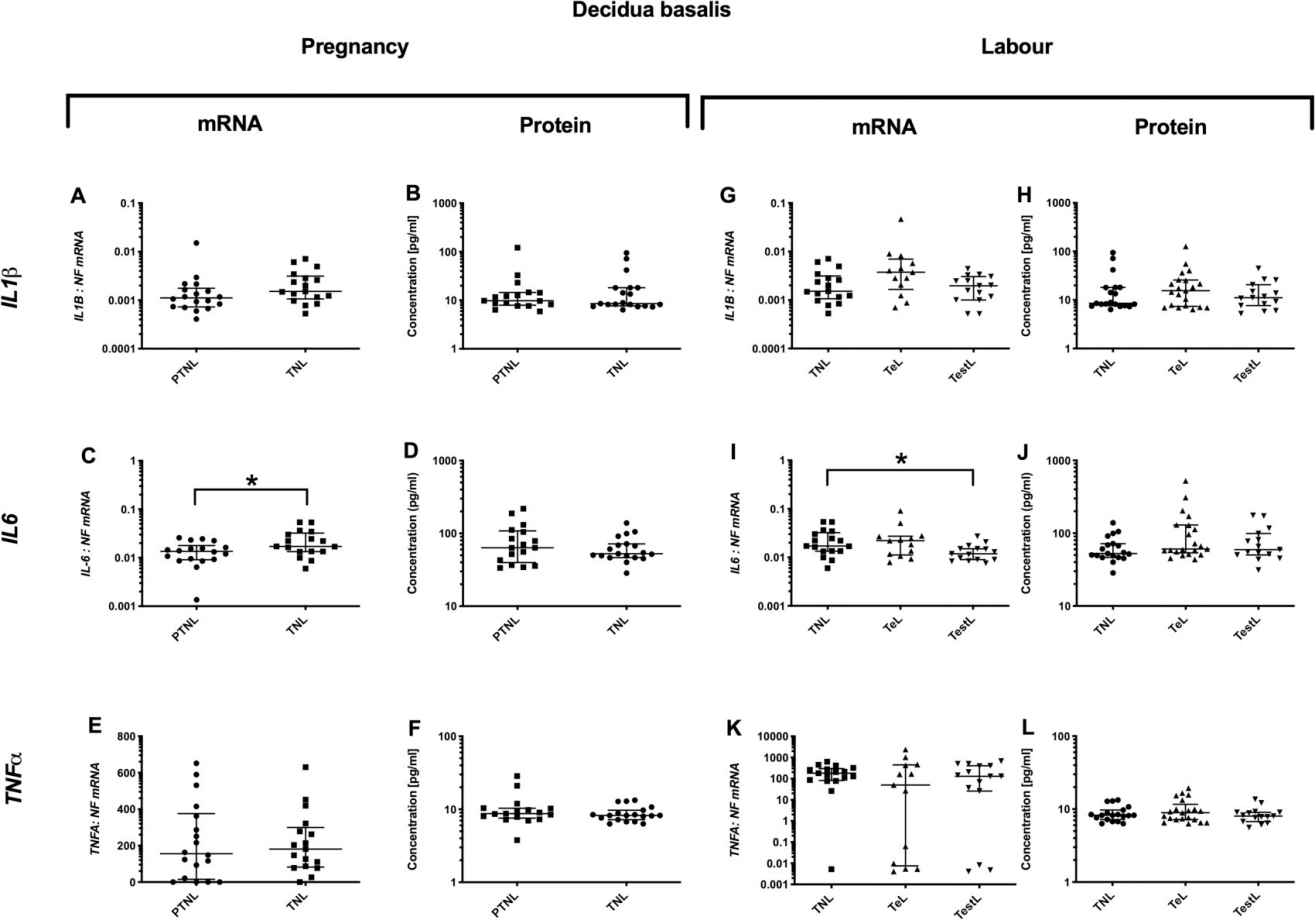
Pro-inflammatory cytokine changes in the decidua basalis. Cytokine levels in the decidual basalis tissues from four groups of women (mean gestational age ± SD in each case), PTNL (33.8±1.7weeks, n = 17), TNL (39.3 ±0.8 weeks, n = 19), TeL (38.4±1.3weeks, n = 21) and TestL (39.5±1.0, n = 15). Tissue levels of the cytokines were quantified using Bio-plex human cytokine 19-plex-array kit (Bio-rad) as describe in *Materials and Methods*. A subset of amnion samples, PTNL (33.8±1.7 weeks, n = 17), TNL (39.3 ±0.9 weeks, n = 16), TeL (38.6±1.2 weeks, n = 13) and TestL (39.5±1.0, n = 15) were used to compare the mRNA expression using rtPCR of *IL-1*β, *IL6 and TNFα* mRNA. Normally distributed data were analysed using a Student’s t-test for two groups and an ANOVA followed by a Dunnett’s or Bonferroni’s post-hoc test for three groups or more. Data that were not normally distributed were analysed using a Mann Whitney test for 2 groups and when comparing three groups or more a Friedman’s test, with a Dunn’s multiple comparisons post-hoc test. The data are shown as median with interquartile range. The p values are demonstrated by * is p<0.05 and ** is p<0.01.

**Fig 5:**
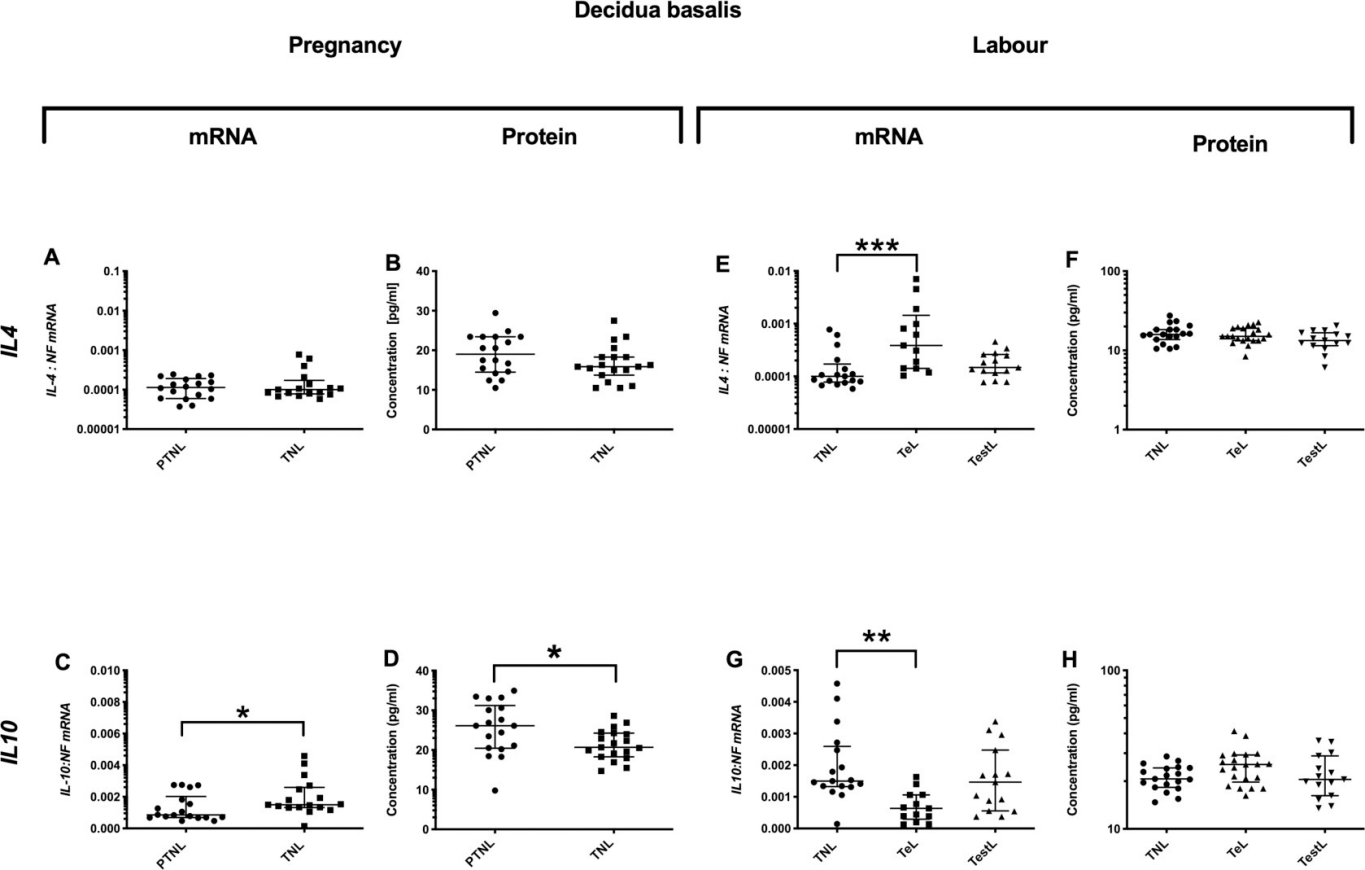
Anti-inflammatory cytokine changes in the decidua basalis. Cytokine levels in the decidual basalis tissues from four groups of women (mean gestational age ± SD in each case), PTNL (33.8±1.7weeks, n = 17), TNL (39.3 ±0.8 weeks, n = 19), TeL (38.4±1.3weeks, n = 21) and TestL (39.5±1.0, n = 15). Tissue levels of the cytokines were quantified using Bio-plex human cytokine 19-plex-array kit (Bio-rad) as describe in *Materials and Methods*. A subset of amnion samples, PTNL (33.8±1.7 weeks, n = 17), TNL (39.3 ±0.9 weeks, n = 16), TeL (38.6±1.2 weeks, n = 13) and TestL (39.5±1.0, n = 15) were used to compare the mRNA expression using rtPCR of *IL4 and IL10*. Normally distributed data were analysed using a Student’s t-test and for data not normally distributed were analysed using a Mann Whitney test. The data are shown as median with interquartile range. The p values are demonstrated by * is p<0.05 and ** is p<0.01.

**Fig 6:**
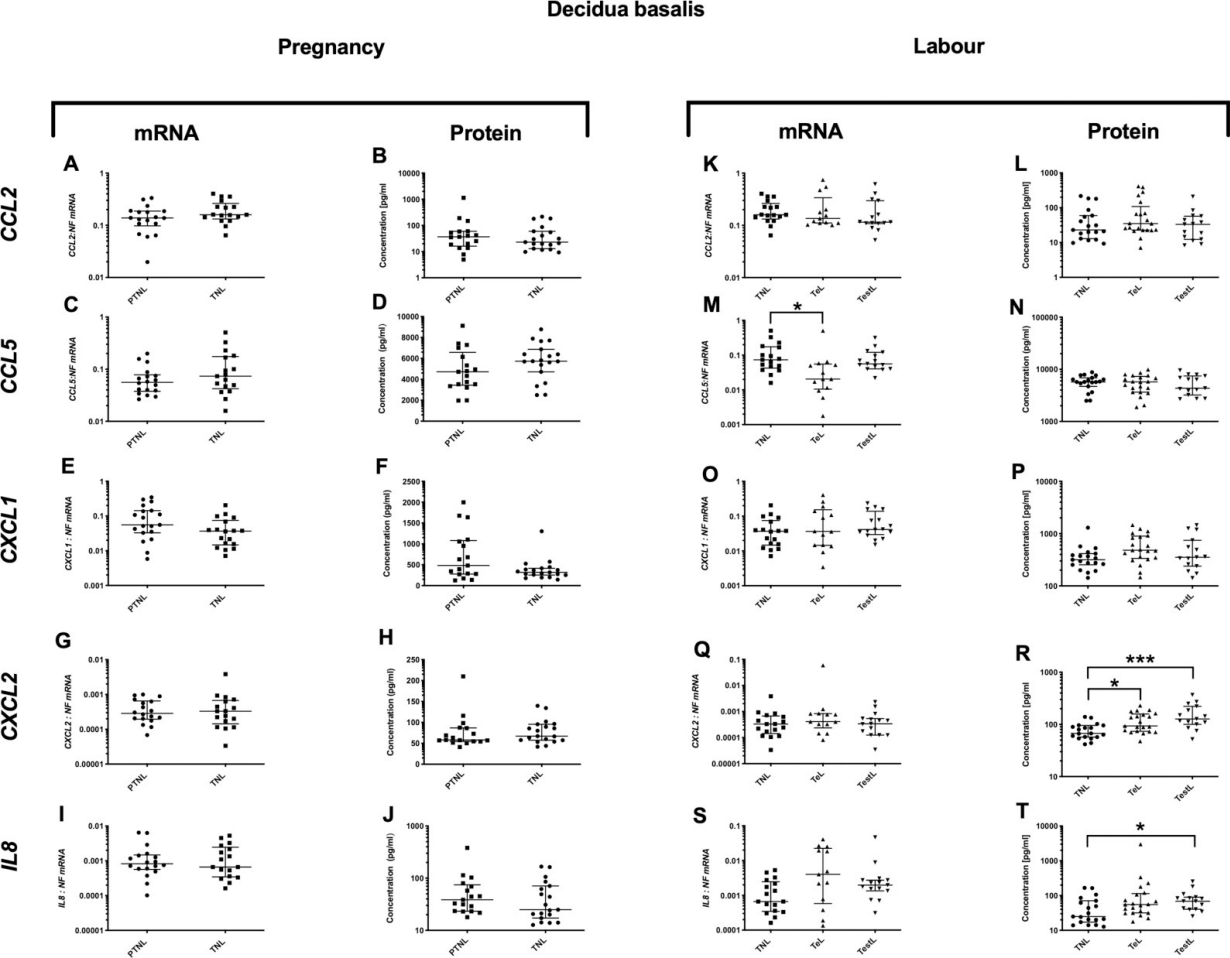
Chemotactic cytokine changes in the decidua basalis. Cytokine levels in the decidual basalis tissues from four groups of women (mean gestational age ± SD in each case), PTNL (33.8±1.7weeks, n = 17), TNL (39.3 ±0.8 weeks, n = 19), TeL (38.4±1.3weeks, n = 21) and TestL (39.5±1.0, n = 15). Tissue levels of the cytokines were quantified using Bio-plex human cytokine 19-plex-array kit (Bio-rad) and a separate assay for *CCL5* as describe in *Materials and Methods*. A subset of amnion samples, PTNL (33.8±1.7 weeks, n = 17), TNL (39.3 ±0.9 weeks, n = 16), TeL (38.6±1.2 weeks, n = 13) and TestL (39.5±1.0, n = 15) were used to compare the mRNA expression using rtPCR of *CCL2*, *CCL5*, *CXCL1*, *CXCL2 and IL-8*. Normally distributed data were analysed using a Student’s t-test for two groups and an ANOVA followed by a Dunnett’s or Bonferroni’s post-hoc test for three groups or more. Data that were not normally distributed were analysed using a Mann Whitney test for 2 groups and when comparing three groups or more a Friedman’s test, with a Dunn’s multiple comparisons post-hoc test. The data are shown as median with interquartile range. The p values are demonstrated by * is p<0.05 and ** is p<0.01.

*Pro-inflammatory changes*. *IL6* mRNA expression was higher in TNL compared to PTNL (p<0.05), but this was not observed in the protein concentrations (Fig 4B). No changes were observed in the mRNA expression or protein concentrations of *IL-1*β and *TNFα* (Fig 4A–4F). *Anti-inflammatory changes*: *IL10* mRNA expression rose with gestation (p<0.05, Fig 5C), but the protein concentrations of *IL10* actually declined in the same samples (p<0.05, Fig 5D). *Chemotactic changes*: No changes were observed in chemokine mRNA and protein with advancing pregnancy (Fig 6A–6J).

#### Labour

*Pro-inflammatory changes*. *IL6* mRNA expression was lower in TestL samples compared to TNL (p<0.05, Fig 4I), but *IL6* protein concentrations were similar across labour (Fig 4J). *Anti-inflammatory changes*: There was an increase in *IL4* mRNA expression, but a decrease in *IL10* (p<0.001 and 0.01, respectively, Fig 5E & 5G). *IL4* and *IL10* protein concentration did not change (Fig 5F & 5H). *Chemotactic changes*: *CCL5* mRNA expression declined (p<0.05, Fig 6M), with no change in the protein concentration (Fig 6N). Although there was no change in *CXCL2* mRNA expression (Fig 6Q), *CXCL2* protein concentrations rose progressively through labour (TNL vs. TeL p<0.05 and TNL vs. TestL p<0.001, Fig 6R). Similarly, although *IL8* mRNA expression did not increase (Fig 6S), *IL8* protein concentration was higher in TestL vs. TNL samples (p<0.05, Fig 6T).

#### Other cytokine changes

*In pregnancy*, no changes were observed; *in labour*, in TestL samples, *CCL25* (p<0.005) protein concentrations was lower compared to TNL (S6 Table).

### Choriodecidua parietalis cytokine profile

#### Pregnancy

**Fig 7:**
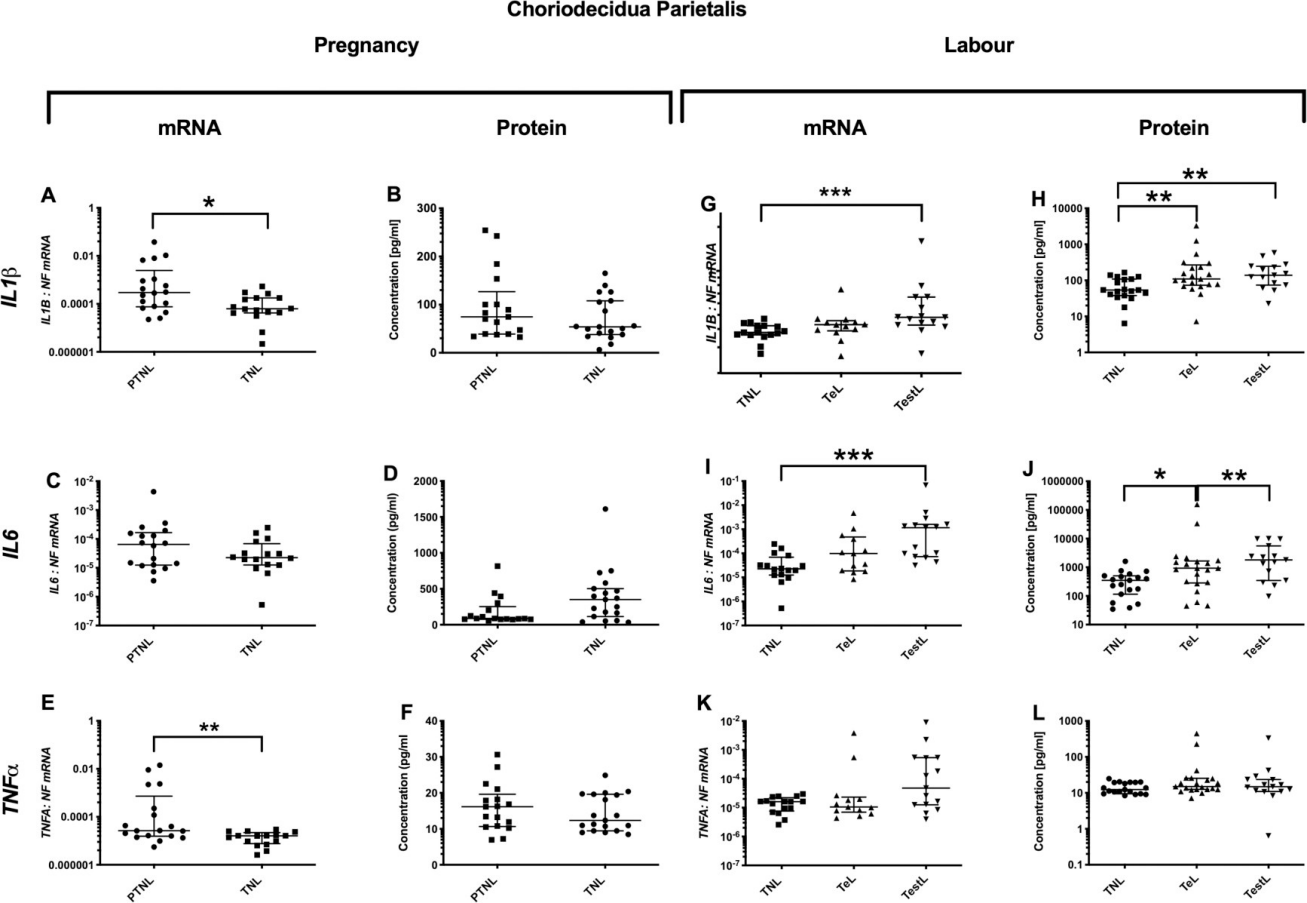
Pro-inflammatory changes in the choriodecidua parietalis. The cytokine levels in the choriodecidua parietalis samples obtained from four groups of women (mean gestational age ± SD in each case), PTNL (33.8±1.7weeks, n = 17), TNL (39.3 ±0.8 weeks, n = 19), TeL (38.4±1.3weeks, n = 21) and TestL (39.5±1.0, n = 15) were compared. Tissue levels of the cytokines were quantified using Bio-plex human cytokine 19-plex-array kit (Bio-rad) and a separate assay for *CCL5* as describe in *Materials and Methods*. A subset of choriodecidua parietalis samples, PTNL (33.8±1.7 weeks, n = 17), TNL (39.3 ±0.9 weeks, n = 16), TeL (38.6±1.2 weeks, n = 13) and TestL (39.5±1.0, n = 15) were used to compare the mRNA expression using rtPCR of *IL-1*β, *IL6* and *TNFα*. Normally distributed data were analysed using a Student’s t-test for two groups and an ANOVA followed by a Dunnett’s or Bonferroni’s post-hoc test for three groups or more. Data that were not normally distributed were analysed using a Mann Whitney test for 2 groups and when comparing three groups or more a Friedman’s test, with a Dunn’s multiple comparisons post-hoc test. The data are shown as median with interquartile range. The p values are demonstrated by * is p<0.05 and ** is p<0.01.

**Fig 8:**
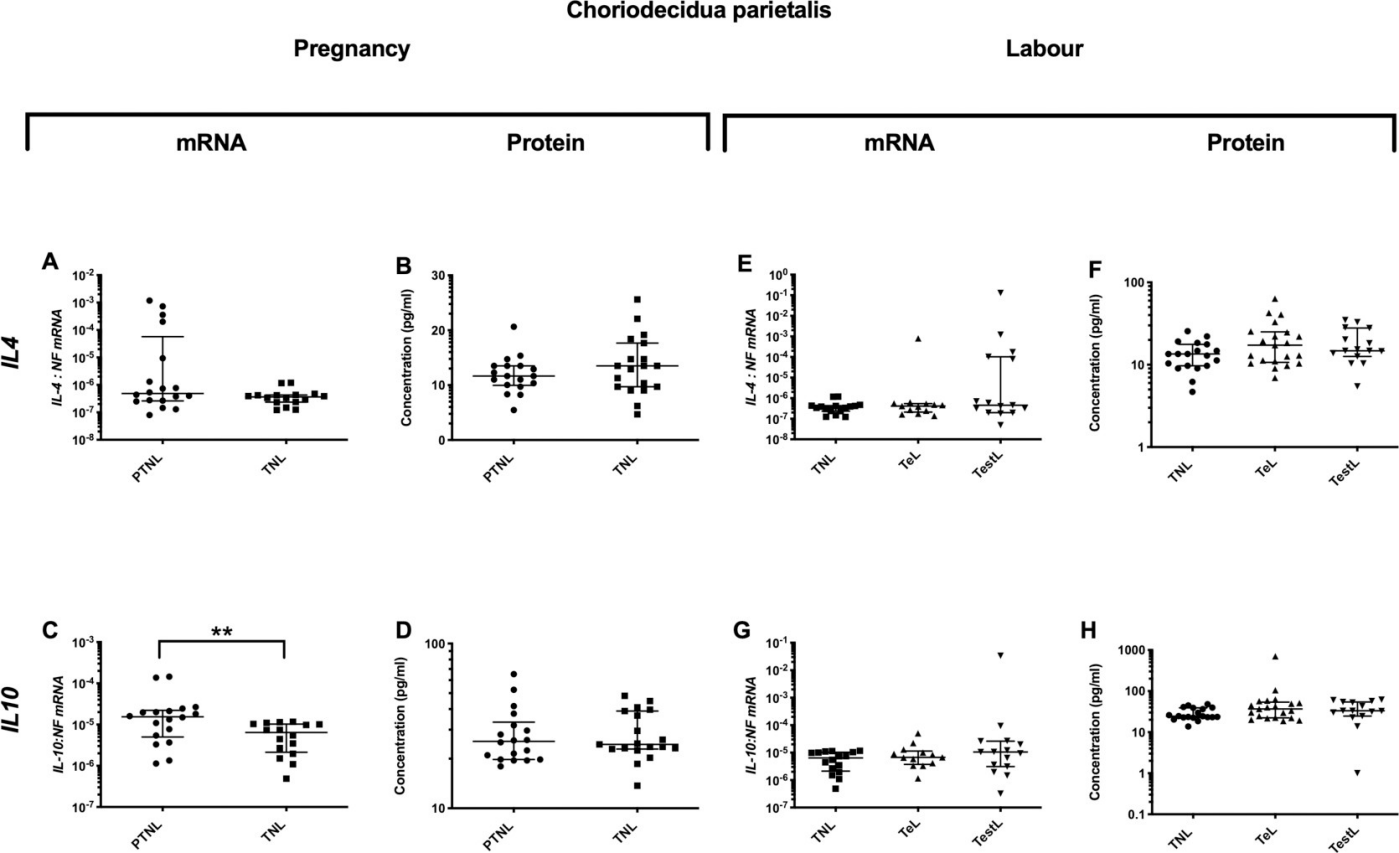
Anti-inflammatory changes in the choriodecidua parietalis. The cytokine levels in the choriodecidua parietalis samples obtained from four groups of women (mean gestational age ± SD in each case), PTNL (33.8±1.7weeks, n = 17), TNL (39.3 ±0.8 weeks, n = 19), TeL (38.4±1.3weeks, n = 21) and TestL (39.5±1.0, n = 15) were compared. Tissue levels of the cytokines were quantified using Bio-plex human cytokine 19-plex-array kit (Bio-rad) and a separate assay for *CCL5* as describe in *Materials and Methods*. A subset of choriodecidua parietalis samples, PTNL (33.8±1.7 weeks, n = 17), TNL (39.3 ±0.9 weeks, n = 16), TeL (38.6±1.2 weeks, n = 13) and TestL (39.5±1.0, n = 15) were used to compare the mRNA expression using rtPCR of *IL4 and IL10*. Normally distributed data were analysed using a Student’s t-test and data not normally distributed were analysed using a Mann Whitney test. The data are shown as median with interquartile range. The p values are demonstrated by * is p<0.05 and ** is p<0.01.

**Fig 9:**
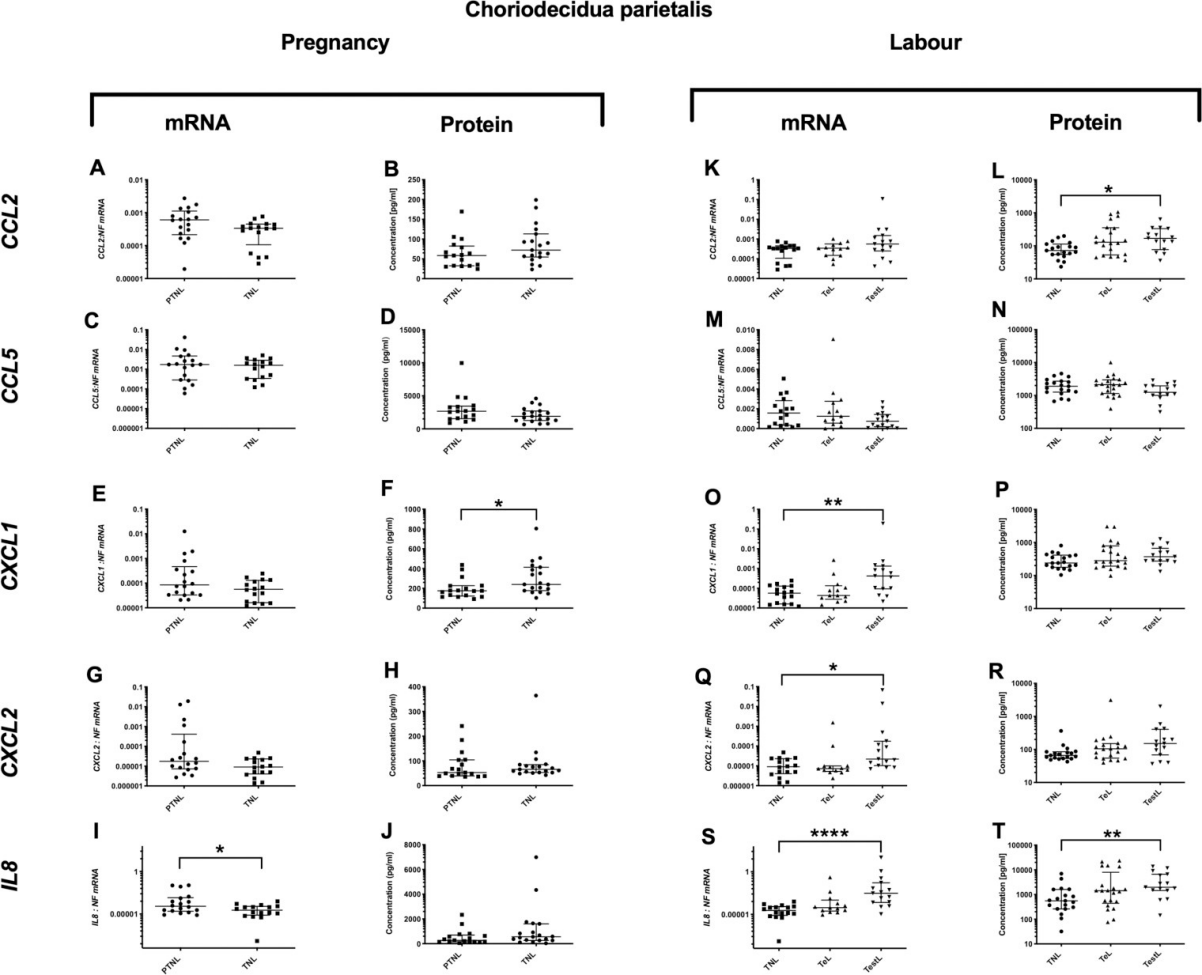
Chemotactic cytokine changes in the choriodecidua parietalis. The cytokine levels in the choriodecidua parietalis samples obtained from four groups of women (mean gestational age ± SD in each case), PTNL (33.8±1.7weeks, n = 17), TNL (39.3 ±0.8 weeks, n = 19), TeL (38.4±1.3weeks, n = 21) and TestL (39.5±1.0, n = 15) were compared. Tissue levels of the cytokines were quantified using Bio-plex human cytokine 19-plex-array kit (Bio-rad) and a separate assay for *CCL5* as describe in *Materials and Methods*. A subset of choriodecidua parietalis samples, PTNL (33.8±1.7 weeks, n = 17), TNL (39.3 ±0.9 weeks, n = 16), TeL (38.6±1.2 weeks, n = 13) and TestL (39.5±1.0, n = 15) were used to compare the mRNA expression using rtPCR of *CCL2*, *CCL5*, *CXCL1*, *CXCL2 and IL-8*. Normally distributed data were analysed using a Student’s t-test for two groups and an ANOVA followed by a Dunnett’s or Bonferroni’s post-hoc test for three groups or more. Data that were not normally distributed were analysed using a Mann Whitney test for 2 groups and when comparing three groups or more a Friedman’s test, with a Dunn’s multiple comparisons post-hoc test. The data are shown as median with interquartile range. The p values are demonstrated by * is p<0.05 and ** is p<0.01.

*Pro-inflammatory changes*. Although *IL-1*β and *TNFα* mRNA expression decreased (p<0.05 and <0.01 respectively (Fig 7A & 7C), there was no change in the protein concentration (Fig 7B & 7F). *Anti-inflammatory changes*: *IL10* mRNA expression decreased (p<0.01, but again there was no change in *IL10* protein concentration (Fig 8C and 8D). *Chemotactic changes*: Similarly, *IL8* mRNA expression decreased (p<0.05, Fig 9I), but *IL8* protein concentration did not change (Fig 9J). Conversely, *CXCL1* protein increased without any change in mRNA expression (p<0.05, Fig 9E & 9F).

#### Labour

*Pro-inflammatory changes*. The mRNA expressions of *IL-1*β and *IL6* rose in TestL samples (p<0.001 for both, Fig 7G & 7I). For both *IL1B* and *IL6*, the protein concentrations were higher in both TeL and TestL compared to TNL samples (for *IL1B* p<0.01 and p<0.01 and for *IL6* p<0.05 and p<0.01 respectively, Fig 7H & 7J). *Anti-inflammatory changes*: No changes were observed during labour in the mRNA or protein levels of *IL4* and IL10 (Fig 8E–8H). *Chemotactic changes*: The mRNA expressions of CXCL1, CXCL2 and *IL8* increased (TNL vs. TestL, p<0.01, <0.05 and <0.0001 respectively (Fig 9O, 9Q & 9S). In terms of chemokine protein concentration, only CCL2 and *IL8* were higher in TestL samples compared to TNL samples (p<0.05 and <0.01 respectively, Fig 9L & 9T).

#### Other cytokine changes

*In pregnancy*, no changes were observed; *in labour*: Interestingly the levels *IL2*, *IFN-γ*, *CCL1*, *CCL25* and *CCL1*7 were higher in TeL compared to TNL samples (p<0.05–0.001, S7 Table) and *IFN-γ* and *CCL25* were higher in TestL compared to TNL (p<0.05 and P<0.01 respectively, S7 Table).

#### The relative change in cytokine levels across tissues

**Fig 10:**
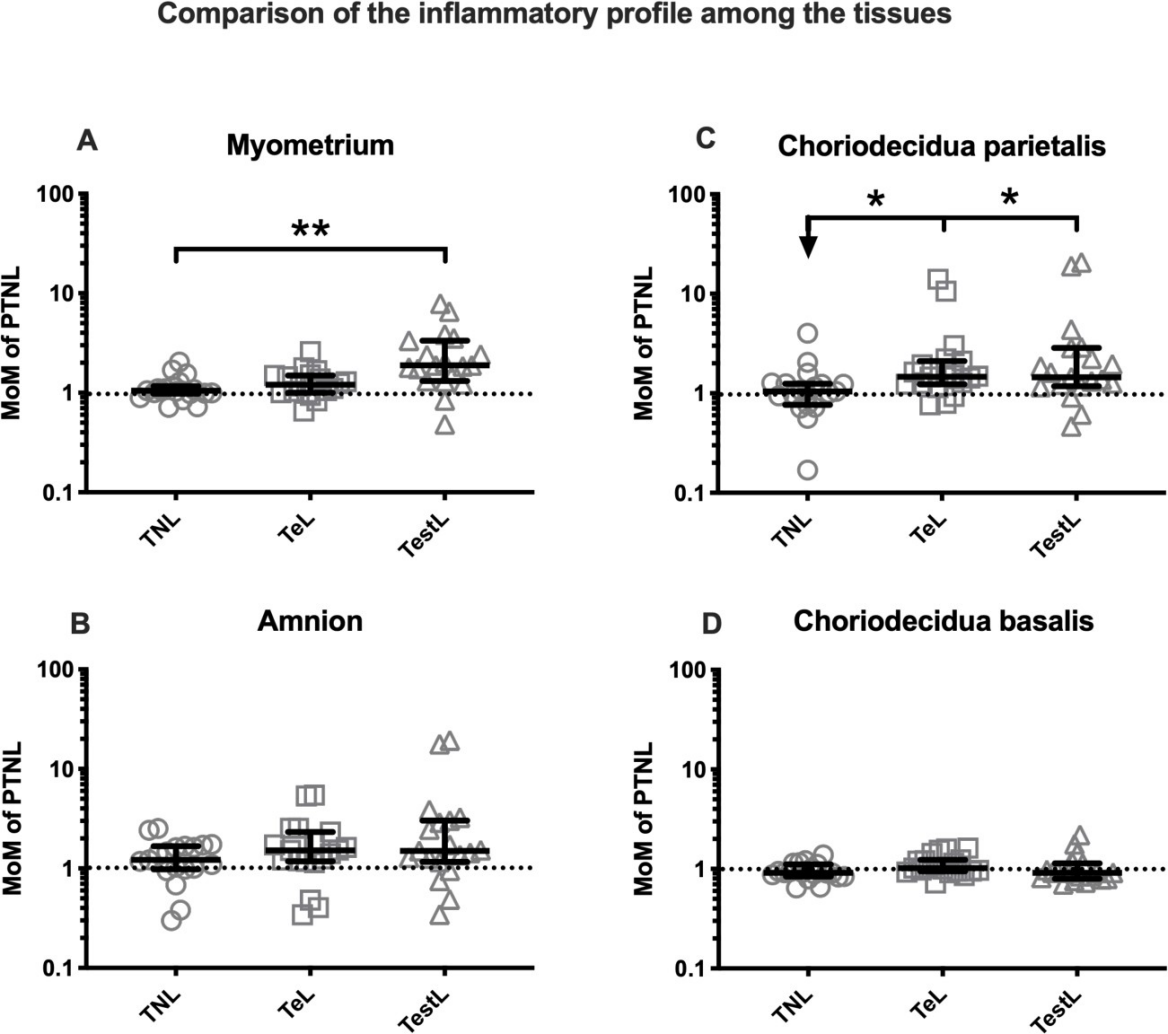
Comparison of the inflammatory changes in the tissues during pregnancy and labour. The multiples of median (MOM) were used to compare the inflammatory changes during gestation, early labour and established labour in each tissue. The median value of PTNL was used to normalise the data across the comparison groups (TNL, TeL and TestL). The median ranges were then used for all the 20 cytokines and comparisons between 2 or more groups were performed using the Kruksal-Wallis test with a Dunn’s Multiple Comparison post hoc test as the data was non-parametric. In the graphs, the p values are demonstrated by * where * is p<0.05, ** is p<0.001 and *** is p<0.0001.

To compare how the overall levels of cytokines differ across the tissues throughout pregnancy and with labour, we calculated the multiple of median (MoM) of the cytokines (the median cytokine concentration of the cytokines from the TNL, TeL and TestL samples by the median of the PTNL samples) from the following tissues: myometrium, amnion, choriodecidua parietalis and decidua basalis (Fig 10A–10D).

With labour, in myometrium only the TestL MoM were greater than TNL MoM (p<0.01, Fig 10A), in the choriodecidua parietalis, both TeL and TestL MoM were greater than TNL MoM (p<0.05 for both, Fig 10C). There were no differences for amnion or decidua basalis (Fig 10B & 10D).

### Transcription factor activation in the choriodecidua parietalis

Since observed greater changes in cytokine levels in the choriodecidua parietalis, we assessed the activity of the *NFKB* and *MAPK/AP1* pathways in this tissue. There were no significant changes in the binding of both *NFKB*, *AP1* and *Phospho-CREB* (S1 Fig).

### PGHS2, CX43, OT, OTR mRNA expression

To test the hypothesis that inflammation in the choriodecidua parietalis drives the onset of labour, we assessed whether there was any change in the mRNA levels of the prolabour genes, reasoning that multiple papers have shown that inflammation increases the levels of *OTR*, *PGHS2* and other prostaglandin synthetic enzymes and/or reduces the levels of the prostaglandin dehydrogenase (*PGDH*) [26]. However, there was no change in the mRNA expression of any of the selected prolabour genes in the choriodecidua parietalis (S2 and S3 Figs). In the decidua basalis, the only change observed was an increase in the *OTR* mRNA expression at the onset of labour (S3 Fig). We did not observe any changes in the mRNA expression of *OT* and the other prolabour genes in the placenta.

## Discussion

Inflammation is held to have a central role in the onset of preterm and term labour. In our recent paper, we found that myometrial inflammation was most likely to be a consequence rather than a cause of labour [8]. Here, we tested the hypothesis that inflammation in other reproductive tissues may drive the onset of term labour. We found that inflammation was present in choriodecidual parietalis in early labour samples, suggesting that choriodecidual inflammation may drive the onset of labour. Several themes emerged from the results, the frequent lack of correlation between mRNA and protein levels; the lack of inflammation in the amnion and placenta; and despite the presence of choriodecidual inflammation, the absence of either transcription factor activation or prolabour gene expression, in contrast with our observations in human myometrium [8, 27].

### The effect of pregnancy in each tissue

#### Amnion

Parry et al. was one of the first to show that as the pregnancy advances the expansion of the uterus is not accompanied by increased growth of the amnion, rather the amnion is passively stretched [28]. Menon showed that as the pregnancy grows, the fetal membranes undergo telomere dependent aging, developing a senescence-associated secretory phenotype (SASP), culminating in the release of a range of factors including cytokines, chemokines and damage associated molecular pattern (DAMPs) markers, which could drive the onset of labour [29]. Indeed, *TNFα* levels have been shown to increase in the amniotic fluid as pregnancy advances [30] and possibly to contribute to membrane weakness through a process involving collagen remodeling and apoptosis [31]. Indeed, *in vitro* work has shown that mechanical stretch is associated with an up-regulation of stretch responsive cytokines such as *IL8* in amniotic cells [32] and increased mRNA expression in amniotic cells of *IL-1*β which is associated with a *NFKB* activation and downstream increase in COX-2 and PGE_2_ synthesis [9]. mRNA expression of *IL8* have been shown to peak after 2 hrs of static stretching of primary amniotic epithelial cells after which levels decline [33]. Others also observed that the transient increase in the mRNA expression of *IL8* found when the amniotic cells are stretched did not translate into protein [34]. However, we found no evidence of a consistent increase in the cytokine levels in amniotic tissue. It is possible that amniotic fluid cytokines are fetal in origin, present in fetal urine, rather than being released from the amnion, explaining the discrepancy. We did observe an increase in *IL10* in the TNL samples compared to the PTNL samples, which would repress inflammation, consistent with the decline in cytokine mRNA levels we observed. Consistent with the *in vitro* observation of a stretch induced increase in prostaglandin synthesis, the amnion is held to be the major source of prostaglandin synthesis [35] Recent papers have suggested that the amnion may show a pre-labour activation at term [36]. In this study, the authors found that amnion with higher *NFKB* activation expressed more *PGHS2*, *IL8* and OTR mRNA [36]. In contrast, we found no evidence of any increase in prolabour or inflammatory gene expression with advancing pregnancy, quite the opposite, the mRNA levels of *TNFα*, *IL4*, *IL8*, *CCL2* and *CXCL2* actually declined, although these changes were not associated with a change in protein levels. Our data suggest that there is no preterm/prelabour activation of the amnion. Further, we explored prolabour gene expression and found no increase in the mRNA expression supporting the assertion that there is no amnion activation in late pregnancy. In the multiplex assay analysis, we did find that *CCL1* and *CCL20* increased with advancing pregnancy. The *CCL20* data are consistent with the report that amniotic fluid concentrations of *CCL20* increase towards term [37], but *CCL1* has not been studied before although, *in vitro*, primary amnion cells have been shown to secrete a variety of cytokines [38–41]. *CCL1* and CCL20 are both chemotactic for monocytes, immature B cells and dendritic cells, which may promote inflammation, weakening the fetal membranes leading to rupture of membranes. Alternatively, *CCL20* has antimicrobial properties [42] and may contribute to the development of a sterile environment for the developing fetus.

#### Decidua basalis

During pregnancy, the mRNA levels of *IL6* and *IL10* increased, but *IL6* protein levels were unchanged and those of *IL10* reduced. Most of the PTNL subjects had pre-eclampsia and/or intra-uterine growth restriction, conditions which are associated with increased inflammation [43]. In contrast, the TNL group were exclusively made up of uncomplicated pregnancies. The lack of an increase in inflammatory cytokines in the PTNL decidual basalis samples is important as it implies that other PTNL tissues are unlikely to exhibit greater inflammation as a consequence of the underlying diagnosis (PET/IUGR). In the remainder of the multiplex data, only *CX3CL1* levels declined, *CX3CL1* is expressed in the apical microvillus of plasma membrane of the syncytiotrophoblast [44] and apart from being chemotactic for NK cells, monocytes and T cells, it plays an important role in the development and implantation of trophoblastic development and implantation. Placenta *CX3CL1* synthesis is increased in severe pre-eclampsia and by inflammatory cytokines *in vitro* [45, 46], perhaps explaining why we observed a decline in cytokine levels between PTNL and TNL samples.

#### Choriodecidua parietalis

There was a decrease in the mRNA levels of *TNFα*, *IL10* and *IL8* with advancing gestation, but none of these changes were associated with a decline in protein levels. In terms of other cytokines, measured in the multiplex assay, only *CCL20* declined suggesting that there is no consistent increase or decrease in choriodecidual parietalis inflammation with advancing gestation.

### The cytokine changes in the tissues in labour

#### Amnion

The only change in early labour was the increase in *CCL2* mRNA levels, protein levels were increased with established labour. Protein levels of *IL6* and *IL8* increased in TestL samples, similar to our observations in myometrium [8], and suggesting that amnion inflammation is a consequence and not a cause of labour. *In vitro* work with primary epithelial amniotic cells has shown that in contrast to static stretch, cyclical stretch is associated with a 15-fold increase in *IL8* mRNA expression after 4hrs, with a significant increase in *IL8* protein [33]. This would be consistent with our observations, that static stretch, as observed with advancing pregnancy, did not affect *IL8* gene expression, but that cyclical stretch, as observed in labour, did increase *IL8* gene expression. It is possible that static stretch primed amnion cells to respond to subsequent cyclic stretch, but this awaits further study. The increase in *IL8* might promote inflammatory cell infiltration as has been reported in the fetal membranes overlying the presenting part [47] and which may up-regulate cytokine mediated inflammatory changes in the amnion, contributing to membrane rupture during labour. Although *IL10* mRNA levels decreased in TestL samples, *IL10* protein levels did not change.

#### Decidua basalis

Given that the decidua basalis is the maternal-fetal interface, processes to minimise inflammation should dominate. Indeed, decidual cytokines, including *IL10* [48], play a major role in modulating the maternal immune response to the fetal allograft during pregnancy. However, the role of decidual cytokines in the onset of labour is less well defined [49]. The observed decline in placental *IL6* mRNA would be consistent with a process to reduce inflammation, although the decline in *IL6* mRNA was not reflected in a reduction in protein levels. These data are consistent with other studies, [50] but contrast to our observations in other tissues, myometrium [8], amnion and choriodecidua parietalis, where we found an increase in pro-inflammatory cytokine levels in TestL samples. Others have shown that labour seems to be associated with a diminution of the anti-inflammatory *IL10* [51]. In this study, we found that the mRNA levels of anti-inflammatory cytokines, *IL4* and *IL10*, increased and decreased respectively in TeL samples, but with no associated change in protein levels, suggesting, if anything, maintenance of the inflammatory balance with labour onset and progression. For the chemokines, *CCL5* behaved similarly, with a decline in mRNA levels in TeL samples, but no change in protein levels; in contrast, *CXCL2* and *IL8* mRNA levels were unchanged but protein levels increased, for *CXCL2* in both TeL and TestL and for *IL8* only in TestL samples. Whether these changes are associated with an increase inflammatory cell infiltration is debated, our preliminary data showing no change in neutrophil or monocytes numbers (unpublished observation) and others showing that inflammatory cells express increased pro-inflammatory cytokines and MMP9 with labour onset [52], but not quantitating leukocyte numbers.

#### Choriodecidua parietalis

We found increases in *IL-1*β and *IL6* levels in both TeL and TestL samples, raising the possibility that inflammation in the choriodecidua parietalis may precipitate the onset of labour. Anti-inflammatory cytokines did not change and *CCL2* and *IL8* were only increased in TestL samples, suggesting that there was no change in the chemokine gradient before the onset of labour. Although the increase in *IL-1*β and *IL6* is consistent with earlier studies that reported an increase in *IL1B*and other cytokines such as *IL8*, and *TNFα*, in association with leukocyte infiltration (neutrophils, macrophages, monocytes, and T and B lymphocytes) in the choriodecidua parietalis [2, 4, 53]. In addition, *IL1B* has been shown to stimulate the production of *IL8* by the choriodecidua [54], which would enhance the influx of neutrophils, increasing the inflammatory response [55, 56]. The increased inflammatory profile in the choriodecidua parietalis in TeL samples was confirmed in the remainder of the multiplex analysis, with *IL2*, IFN-γ, *CCL1* and *CCL25* being elevated, with IFN-γ and *CCL25* remaining high in TestL samples. In first trimester decidua, IFN-γ is derived from both NK cells and decidual cells [57] and has been implicated in the aetiology of pre-eclampsia [58]. Consistent with the increase in IFN-γ, we have seen greater numbers of NK cells in the choriodecidual of early laboring women (unpublished observation), suggesting that NK cells may have a role both in the increase in choriodecidual inflammation and the onset of labour. Intriguingly, activated NK cells produce *CCL1* and *IL8* amongst other cytokines [59], and we found both to be elevated in labouring choriodecidua. *CCL25*, another chemokine found to be markedly elevated in TeL choriodecidual samples, may regulate NK cell function as subsets of NK cells express its receptor *CCR9* [60]. Similarly, *IL2* has been reported to stimulate both NK cells and T cells, with authors suggesting a role in cancer immunotherapy [61]. Collectively, the profile of cytokine increases in the choriodecidua parietalis in early labour suggests a potential role for increased NK cell activity, which may contribute to the increase in inflammation and possibly the onset of labour.

### Correlation between mRNA and protein

For some cytokines, the mRNA did not necessarily match with the protein data and it is recognised that correlation can be poor [62]. We evaluated the correlation between the mRNA expression and protein level. We found that there was strong to moderate correlation with IL6, IL8, CCL5 and CXCL1 with the range of correlation coefficients of 0.68 to 0.40, and weak correlations with IL1b, TNFa and CXCL2 with the range of correlation coefficients of 0.39 to 0.27, while there was no correlation between mRNA and protein expression for IL4, IL10 and CCL2 (S8 Table). Under steady sate conditions we would normally expect to see a close relationship between mRNA expression and protein levels. However, under normal physiological conditions human tissues will vary in the timing of mRNA translation to protein. It may therefore be possible that at the time the tissues were biopsied translation to proteins did not occur or the peak of the protein expression had occurred earlier. Furthermore, post-transcriptional modifications may have occurred which may explain the differences.

#### Comparative effects of labour in different tissues

We compared the inflammatory signatures of each tissue with the onset of labour by converting the cytokine multiplex data to multiple of the PTNL medians. These data showed that inflammatory changes in TeL samples were most marked in the choriodecidua parietalis. Inflammatory changes TestL were present in both myometrium and choriodecidua parietalis. Interestingly, the decidua basalis displayed minimal inflammatory change with labour, suggesting that inflammation is tightly controlled in this tissue, consistent with its critical role in supporting the growth and development of the fetus.

In the myometrium, we were able to show activation of the *NFKB* system with the onset of labour [8], however we could not detect and increase in either *NFKB*, *AP1* or *pCREB* activity in the choriodecidua parietalis with the onset of labour. This may have been because we missed the increase in transcription factor activation, which may have been even earlier in the labour process and would probably have been a short-lived, acute process.

Next, we investigated whether the inflammation in the choriodecidual parietalis was associated with increased prolabour gene expression. We studied the mRNA levels of *OT*, *PGHS2*, *OTR*, *Cx43* and *PDGH* in the three tissues and failed to show any change with advancing gestation or the onset of labour (S2 and S3 Figs). Our data are similar to earlier studies which did not observe an increase in *OTR* mRNA expression at the onset of labour in the choriodecidual parietalis, amnion or decidua basalis [63–65]. However, our data contrast with many previous reports, which have found that labour is associated with an upregulation of pro-labour genes in the amnion, choriodecidua parietalis and decidual basalis and may be due to the use of established labour rather than early labour samples in their cohort [15, 23, 36, 66, 67], although we did not see any marked increase in established labour samples either.

## Conclusion

Our findings suggest that the gestational compartments work synergistically together during the pregnancy to maintain an anti-inflammatory state by counteracting the inflammatory response to stretch. At the onset of labour, there an increase in the inflammatory cytokines in the choriodecidua parietalis suggesting that the inflammatory signal for the onset of labour originates in the choriodecidua parietalis, and act on the myometrium via the NfKB pathways to drive increase in OTR expression and the onset of labour.

## Supporting information

S1 Table Demographic table of the women for the choriodecidua parietalis and decidua basalis tissues used for the multiplex assay. (DOCX)

S2 Table Demographic table of the women for the amnion tissues used in the multiplex assay. (DOCX)

S3 Table List of Bio-Plex® analytes included in the custom-made Bio-Plex® 19-plex^TM^ assay. (DOCX)

S4 Table All mRNA genes used for PCR including: Forward and reverse primer sequences, genbank/accession number and base pair lengths. (DOCX)

S5 Table Summary of cytokine concentrations in amnion. (DOCX)

S6 Table Summary of cytokine concentrations in decidua basalis. (DOCX)

S7 Table Summary of cytokine concentrations in choriodecidua parietalis. (DOCX)

S8 Table Correlation of mRNA and protein. (DOCX)

S1 Fig Transcription factor activation in the choriodecidua parietalis. Choriodecidua parietalis samples were obtained from four groups of women at the time of Caesarean section from women (mean gestational age ± SD in each case), at preterm no labour (PTNL; 33.2 ± 2.5 weeks, n = 18), term no labour (TNL; 39.5 ± 0.8 weeks, n = 19), early labour (38.3 ± 1.1 weeks, n = 18) and term established labour (39.5 ± 1.0, n = 15). The samples were homogenised and relative levels of *Phospho-cJun*, *Phospho-NFKB p65* and *Phospho-CREB* were measured using TransAMTM *NFKB* and TransAMTM *AP1* transcription factor DNA-protein binding assays (Active Motif, Carlsbad CA, USA). Normally distributed data were analysed using a student’s t-test for two groups and an ANOVA followed by a Dunnett’s or Bonferroni’s post-hoc test for three groups or more. Data that were not normally distributed were analysed using a Mann Whitney test for 2 groups and when comparing three groups or more a Friedman’s test, with a Dunn’s multiple comparisons post-hoc test. The data are shown as median with interquartile range. The p values are demonstrated by * is p<0.05, ** is p<0.01 and **** is p<0.0001.
(TIFF)

S2 Fig Pro-labour gene expression in the tissues. A subset of amnion, choriodecidual parietalis and decidua basalis samples were used for mRNA extraction. The samples were homogenised and RNA extracted and converted to cDNA. Copy numbers of PGHS-2, *PGHD*, *OTR* and *CX43* mRNA for term no labour, term early and term established labour samples were measured quantitative rtPCR. Normally distributed data were analysed using an ANOVA followed by a Dunnett’s or Bonferroni’s post-hoc test for three groups or more. Data that were not normally distributed were analysed using a Friedman’s test, with a Dunn’s multiple comparisons post-hoc test. The data are shown as median with interquartile range. The p values are demonstrated by * is p<0.05 and ** is p<0.01.
(TIFF)

S3 Fig Oxytocin expression in the choriodecidua parietalis and decidua basalis. A subset of, choriodecidua parietalis and decidua basalis samples were used for mRNA extraction. Choriodecidua parietalis: term no labour (TNL; 39.3 ± 0.9 weeks, n = 16), early labour (38.3 ± 1.1 weeks, n = 13) and term established labour (39.5 ± 1.0, n = 15). Decidua basalis: term no labour (TNL; 39.3 ± 0.9 weeks, n = 17), early labour (38.7 ± 1.3 weeks, n = 12) and term established labour (39.5 ± 1.0, n = 15). The samples were homogenised and RNA extracted and converted to cDNA. Copy numbers of OT mRNA for the term no labour, term early and term established labour samples were measured quantitative rtPCR. Normally distributed data were analysed using an ANOVA followed by a Dunnett’s or Bonferroni’s post-hoc test for three groups or more. Data that were not normally distributed were analysed using a Friedman’s test, with a Dunn’s multiple comparisons post-hoc test. The data are shown as median with interquartile range. The p values are demonstrated by * is p<0.05 and ** is p<0.01.
(TIFF)
