# Supplementary material for: Is there an inflammatory stimulus to human term labour?
Source: PLoS One. 2021 Aug 31;16(8):e0256545. doi: 10.1371/journal.pone.0256545 (PMC8407546; doi:10.1371/journal.pone.0256545)
Supplement: S1 Table — (DOCX) [file pone.0256545.s001.docx]

S1 Table Demographic table of the women for the choriodecidua parietalis and decidua basalis tissues used for the multiplex assay

| Choriodecidua parietalis and decidua basalis | Preterm no labour  n=17 | Term no labour n=19 | Term early labour n=21 | Term established labour n=15 |
| --- | --- | --- | --- | --- |
| Maternal age | 32±6 | 34±6 | 35±4 | 36±3 |
| Parity |  |  |  |  |
| 0 | 12 | 3 | 12 | 4 |
| 1 | 3 | 12 | 8 | 8 |
| 2 | 2 | 3 | 1 | 1 |
| 3 |  | 0 | 0 | 1 |
| 4 |  | 1 |  |  |
| BMI | 26.5±4.8 | 25.5±4.8 | 22.7±2.8 | 22.6±3.2 |
| Gestational age (mean ±SD) | 33.8±1.7 | 39.3 ±0.8 | 38.4±1.3 | 39.5±1.0 |
| Indication for Caesarean section (CS) | | | | |
| Preeclampsia  (PET) | 8 |  |  |  |
| PET with fetal growth restriction (IUGR) | 4 |  |  |  |
| Abnormal fetal heart tracing | 2 |  | 4 | 4 |
| Previous ruptured uterus | 1 |  |  |  |
| IUGR | 2 |  |  |  |
| Breech or transverse lie | 0 | 3 | 7 | 7 |
| Previous CS | 0 | 13 | 6 | 2 |
| Maternal request |  | 3 | 4 | 1 |
| Prelabour ruptured membranes | 0 | 0 | 15 | 9 |
| Length of rupture of membranes (hours) | N/A | N/A | 8.2±8.8 | 9.3±9.5 |
