# Supplementary material for: Is there an inflammatory stimulus to human term labour?
Source: PLoS One. 2021 Aug 31;16(8):e0256545. doi: 10.1371/journal.pone.0256545 (PMC8407546; doi:10.1371/journal.pone.0256545)
Supplement: S2 Table — (DOCX) [file pone.0256545.s002.docx]

S2 Table Demographic table of the women for the amnion tissues used in the multiplex assay

| Amnion | Preterm No Labour n=12 | Term no labour n=13 | Term early labour n=15 | Term established labour n=11 |
| --- | --- | --- | --- | --- |
| Maternal age | 32±2 | 32±2 | 34±4 | 36±4 |
| Parity |  |  |  |  |
| 0 | 12 | 2 | 7 | 2 |
| 1 | 3 | 12 | 8 | 7 |
| 2 | 2 | 9 | 0 | 1 |
| 3 |  | 0 | 0 | 1 |
| BMI | 26.5±4.8 | 27.4±7.0 | 22.9±2.6 | 22.3±3.2 |
| Gestational age (mean +-SD) | 33.8±1.7 | 39.3 ±0.9 | 38.6±1.2 | 39.6±1.2 |
| Indication for CS | | | | |
| PET | 3 |  |  |  |
| PET with IUGR | 3 |  |  |  |
| Abnormal fetal heart tracing | 3 |  | 2 | 4 |
| Previous ruptured uterus | 1 |  |  |  |
| IUGR | 2 |  |  |  |
| Breech or transverse lie | 0 | 0 | 4 | 7 |
| Previous CS | 0 | 9 | 4 | 5 |
| Maternal request |  | 4 | 5 | 0 |
| Prelabour rupture of membranes | 0 | 0 | 12 | 7 |
| Average length of time of rupture of membranes | N/A | N/A | 8.1±8.3 | 10.3±9.0 |
