# Supplementary material for: Is there an inflammatory stimulus to human term labour?
Source: PLoS One. 2021 Aug 31;16(8):e0256545. doi: 10.1371/journal.pone.0256545 (PMC8407546; doi:10.1371/journal.pone.0256545)
Supplement: S3 Table — (DOCX) [file pone.0256545.s003.docx]

S3 Table List of Bio-Plex® analytes included in the custom-made Bio-Plex® 19-plex^TM^ assay.

| Bio-Plex® Analytes | | | |
| --- | --- | --- | --- |
| *IL-1*β | *IL10* | *CXCL1* | *CCL20* |
| *IL2* | *IL16* | *CXCL2* | *CCL17* |
| *IL4* | *IFN-γ* | *CXCL6* | *CCL25* |
| *IL6* | *TNFα* | *CCL1* | *CCL2* |
| *IL8* | *CX3CL1* | *CCL7* | *CCL5** |

*CCL5* (*) was analysed in a separate assay due to buffer incompatibility.
