# Supplementary material for: Is there an inflammatory stimulus to human term labour?
Source: PLoS One. 2021 Aug 31;16(8):e0256545. doi: 10.1371/journal.pone.0256545 (PMC8407546; doi:10.1371/journal.pone.0256545)
Supplement: S4 Table — (DOCX) [file pone.0256545.s004.docx]

S4 Table All mRNA genes used for PCR including: forward and reverse primer sequences, genbank/accession number and base pair lengths.

| **Genes** | **Forward Primer sequence** | **Reverse Primer sequence** | **Genbank/**  **EMBL Accession no.** | **Nucleotide base pairs** |
| --- | --- | --- | --- | --- |
| *GAPDH* | 5’-TGATGACATCAAGAAGGTGGTGAAG-3’ | 5’-TCCTTGGAGGCCATGTAGGCCAT-3’ | BC014085 | 239 |
| *18S* | 5’-AAACGGCTACCACATCCAAG-3’ | 5’- CCTCCAATGGATCCTCGTTA -3’ | M10098 | 155 |
| *CYC* | 5’-CGGTGGCAAATTCAAGTCCT-3’ | 5’-TTCCTGAATTCTCCGGTGCA-3’ | AF022115.1 | 247 |
| *IL4* | 5’-tgaacagcctcacagagcag-3’ | 5’-gcgagtgtccttctcatggt-3’ | M13982 | 151 |
| *IL6* | 5’-CCTTCCAAAGATGGCTGAAA-3’ | 5’-AGCTCTGGCTTGTTCCTCAC-3’ | NM000600 | 153 |
| *I-8* | 5’- GCCTTCCTGATTTCTGCAGC -3’ | 5’- CGCAGTGTGGTCCACTCTCA -3’ | NM000584 | 149 |
| *IL10* | 5’-tgccttcagcagagtgaaga-3’ | 5’-ggtcttggttctcagcttgg-3’ | AY029171 | 169 |
| *CXCL1* | 5’-GAAAGCTTGCCTCAATCCTG-3’ | 5’-GCCTCTGCAGCTGTGTCTCT-3’ | NM001511 | 173 |
| *CXCL2* | 5’-ctgctcctgctcctggtg-3’ | 5’-gctttctgcccattcttgag-3’ | NM002089 | 190 |
| *CCL2* | 5’-TCTGTGCCTGCTGCTCATAG-3’ | 5’-AGATCTCCTTGGCCACAATG-3’ | X14768 | 202 |
| *CCL5* | 5’-CCATATTCCTCGGACACCAC-3’ | 5’-TGTACTCCCGAACCCATTTC-3’ | NM002985 | 180 |
| *TNFα* | 5’-GAGAAGGGTGACCGACTCAG-3’ | 5’-GGTTGAGGGTGTCTGAAGGA-3’ | NM000594 | 175 |
| *OTR* | 5_-AGAAGCACTCGCGCCTCTT-3 | 5_-AGGTGATGTCCCACAGCAACT-3 | NM000916 | 101 |
| *Cx43* | 5’-TGGATTCAGCTTGAGTGCTG-3’ | 5’-GGTCGCTCTTTCCCTTAACC-3’ | BC026329 | 205 |
| *PGHS2* | 5’-TGTGCAACACTTGAGTGG CT-3’ | 5’-ACTTTCTGTACTGCGGGTGG-3’ | AY151286 | 296 |
| *PGHD* | 5’-gtgaaggcggcatcattatc-3’ | 5’-ttgattcaaggatggctgtg-3’ | L76465.1 | 198 |
