# Supplementary material for: Is there an inflammatory stimulus to human term labour?
Source: PLoS One. 2021 Aug 31;16(8):e0256545. doi: 10.1371/journal.pone.0256545 (PMC8407546; doi:10.1371/journal.pone.0256545)
Supplement: S5 Table — (DOCX) [file pone.0256545.s005.docx]

S5 Table Summary of cytokine concentrations in Amnion

| **Cytokine** | **PTNL** | | **TNL** | | **TEL** | | **TestL** | |
| --- | --- | --- | --- | --- | --- | --- | --- | --- |
|  | Median | 25th-75th  percentile | Median | 25th-75th  percentile | Median | 25th-75th percentile | Median | 25th-75th percentile |
| IL-2 | 0.36 | 0.285-0.57 | 0.42 | 0.33-0.635 | 0.48 | 0.3-0.73 | 0.57 | 0.33-2.65 |
| IL-16 | 457 | 228.6-567.6 | 188.8 | 122.8-347.9 | 184.3 | 142.3-247.5 | 157.1 | 46.75-273.8 |
| IFNγ | 1.17 | 0.96-2.75 | 1.38 | 1.17-1.995 | 1.79 | 1.17-2.6 | 1.79 | 1.38-11.04 |
| CCL7 | 7.71 | 6.98-12.47 | 8.44 | 7.345-11.37 | 9.17 | 11.37-11.37 | 7.71 | 4.81-37.78 |
| CX3CL1 | 28.22 | 23.81 - 32.31 | 26.78 | 24.94-36.99 | 34.31 | 26.62 -45.19 | 36.15 | 24.21-154.4 |
| CCL1 | 5.3 **^Ψ^** | 5.3-6.9 | 14.68 | 7.895-20.07 | 5.3 | 5.3-13.95 | 11.32 | 5.3-20.36 |
| CCl20 | 4.01 | 3.46-5.345 | 6.7 | 5.22-10.87 | 10.16 | 7.31-18.94 | 12.13 | 8.57-114.9 |
| CCL25 | 44.4 | 33.29-54.83 | 43.52 | 36.41-67.23 | 52.24 | 45.28-67.65 | 51.8 | 35.09-92.88 |
| CXCL6 | 2.96 | 2.09-3.935 | 3.05 | 1.005-6.325 | 5.48 | 4.01-8.9 | 4.24 | 2.37-10.33 |
| CCL17 | Not detected in the amnion | | | | | | | |

*significantly higher compared to TNL, where p<0.05

**^Ψ^** significantly lower compared to TNL where p<0.05
