# Supplementary material for: Is there an inflammatory stimulus to human term labour?
Source: PLoS One. 2021 Aug 31;16(8):e0256545. doi: 10.1371/journal.pone.0256545 (PMC8407546; doi:10.1371/journal.pone.0256545)
Supplement: S6 Table — (DOCX) [file pone.0256545.s006.docx]

S6 Table Summary of cytokine concentrations in decidua basalis

| **Cytokine** | **PTNL** | | **TNL** | | **TEL** | | **Test** | |
| --- | --- | --- | --- | --- | --- | --- | --- | --- |
|  | Median | 25th-75th percentile | Median | 25th-75th percentile | Median | 25th-75th percentile | Median | 25th-75th percentile |
| IL-2 | 1.82 | 1.62-2.06 | 1.62 | 1.48-1.96 | 1.76 | 1.41-2.29 | 1.48 | 1.34-1.89 |
| IL-16 | 1492 | 914.1-1744 | 1354 | 1240-1762 | 1385 | 935.7-1854 | 1268 | 974-1930 |
| IFNγ | 1.14 | 1.015-1.295 | 1.08 | 0.91-1.2 | 1.08 | 0.97-1.31 | 0.91 | 0.73-1.03 |
| CCL7 | 34.86 | 31.05-40.32 | 31.82 | 28.69-37.8 | 31.82 | 31.05-38.88 | 31.82 | 27.08-33.35 |
| CX3CL1 | 115.9 | 90.67-189.6 | 160.8 | 138.1-194.5 | 179.1 | 113-246.7 | 166.6 | 139.5-253.7 |
| CCL1 | 76.21 | 58.34-85.82 | 63.85 | 49.17-78.72 | 55.17 | 49.91-80 | 64.83 | 80-71.58 |
| CCL20 | 6.84 | 5.34-9.655 | 6.28 | 5.41-9.5 | 8.2 | 6.655-11.08 | 6.65 | 5.32-8.02 |
| CCL25 | 200.8 | 187-227.2 | 202.3 | 176.2-243.1 | 205.4 | 183.6-237.7 | 140.8 **^Ψ Ψ^** | 129.8-178.3 |
| CXCL6 | 27.4 | 21.12-36.72 | 30.44 | 23.83-36.89 | 33.72 | 24.41-38.09 | 25.93 | 20.48-33.84 |
| CCL17 | 33.72 | 29.31-40.55 | 38.02 | 28.37-42.45 | 34.27 | 31.42-38.27 | 31.42 | 26.44-38.28 |

**^Ψ^** **^Ψ^** significantly lower compared to TNL where p<0.01
