# Supplementary material for: Is there an inflammatory stimulus to human term labour?
Source: PLoS One. 2021 Aug 31;16(8):e0256545. doi: 10.1371/journal.pone.0256545 (PMC8407546; doi:10.1371/journal.pone.0256545)
Supplement: S7 Table — (DOCX) [file pone.0256545.s007.docx]

S7 Table Summary of cytokine concentrations in choriodecidua parietalis

| **Cytokine** | **PTNL** | | **TNL** | | **TEL** | |  | **TestL** |
| --- | --- | --- | --- | --- | --- | --- | --- | --- |
|  | Median | 25th-75th percentile | Median | 25th-75th percentile | Median | 25th-75th percentile | Median | 25th-75th percentile |
| *IL2* | 2.54 | 2.14-2.990 | 2.54 | 2.04-2.94 | 3.14* | 2.54-4.79 | 2.94 | 2.64-4.3 |
| *IL16* | 1388 | 486.7-1802 | 777.5 | 361.1-1359 | 1062 | 605.4-605.4 | 851.3 | 438-942.7 |
| *IFN-γ* | 1.42 | 1.245-1.775 | 1.48 | 1.08-1.69 | 1.78* | 1.45-2.93 | 1.96* | 1.6-2.87 |
| *CCL7* | 40.12 | 31.45-43.63 | 37.71 | 31.45-44.78 | 45.61 | 37.71-65.07 | 49.29 | 39.31-65.11 |
| *CX3CL1* | 80.91 | 63.05-111.1 | 102.6 | 69.91-122.8 | 132.5 | 96.43-267.7 | 158.3 | 85.45-318.3 |
| *CCL1* | 27.27 | 26.1-34.7 | 29.37 | 25.79-32.55 | 34.8** | 31.63-50.24 | 32.09 | 28.52-39.4 |
| *CCL20* | 13 | 8.525-18.53 | 19.11 | 15.38-34.07 | 39.19 | 18.59-100.3 | 56.87 | 20.31-128.3 |
| *CCL25* | 219.1 | 194.9-255.5 | 210.3 | 199.2-262.9 | 311.9*** | 252.7-502.5 | 318.5** | 244.8-419.1 |
| *CXCL6* | 13.7 | 11.46-17.28 | 16.88 | 13.83-24.15 | 20.89 | 14.68-33.15 | 22.02 | 15.53-30.4 |
| *CCL17* | 23.57 | 20.38-31.99 | 30.48 | 25.05-41.87 | 31.99* | 26.45-44.67 | 26.95 | 23.85-35.69 |

*significantly higher compared to TNL, where p<0.05

** significantly higher compared to TNL, where p<0.01

***significantly higher compared to TNL, where p<0.0001
