# Supplementary material for: Is there an inflammatory stimulus to human term labour?
Source: PLoS One. 2021 Aug 31;16(8):e0256545. doi: 10.1371/journal.pone.0256545 (PMC8407546; doi:10.1371/journal.pone.0256545)
Supplement: S8 Table — (DOCX) [file pone.0256545.s008.docx]

| Cytokine | Spearman coefficient | N | P value |
| --- | --- | --- | --- |
| IL6 | 0.6817 | 22 | <0.0001**** |
| IL8 | 0.4796 | 22 | 0.0002*** |
| IL1b | 0.2748 | 22 | 0.0404* |
| IL4 | 0.0010 | 22 | 0.9944 |
| IL10 | -0.1253 | 22 | 0.3575 |
| TNFa | 0.3870 | 22 | 0.0035** |
| CCL2/MCP-1 | 0.2249 | 22 | 0.0957 |
| CCL5/RANTES | 0.4047 | 22 | 0.0020** |
| CXCL1/GROa | 0.5656 | 22 | <0.0001**** |
| CXCL2/GROb | 0.3251 | 22 | 0.0145* |

S8 Table Correlation between mRNA and protein
