# Supplementary figures and images for: Is there an inflammatory stimulus to human term labour?
Source: PLoS One. 2021 Aug 31;16(8):e0256545. doi: 10.1371/journal.pone.0256545 (PMC8407546; doi:10.1371/journal.pone.0256545)

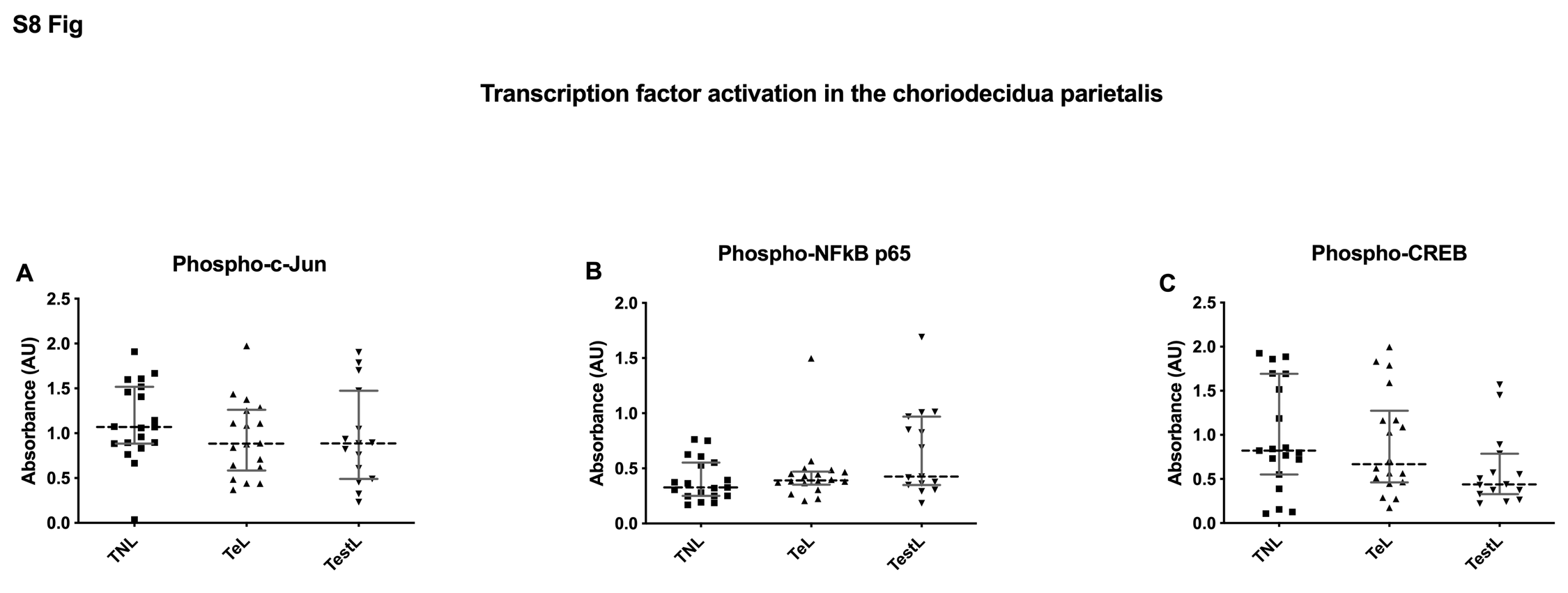

Supplement: S1 Fig — Choriodecidua parietalis samples were obtained from four groups of women at the time of Caesarean section from women (mean gestational age ± SD in each case), at preterm no labour (PTNL; 33.2 ± 2.5 weeks, n = 18), term no labour (TNL; 39.5 ± 0.8 weeks, n = 19), early labour (38.3 ± 1.1 weeks, n = 18) and term established labour (39.5 ± 1.0, n = 15). The samples were homogenised and relative levels of Phospho-cJun, Phospho-NFKB p65 and Phospho-CREB were measured using TransAMTM NFKB and TransAMTM AP1 transcription factor DNA-protein binding assays (Active Motif, Carlsbad CA, USA). Normally distributed data were analysed using a student’s t-test for two groups and an ANOVA followed by a Dunnett’s or Bonferroni’s post-hoc test for three groups or more. Data that were not normally distributed were analysed using a Mann Whitney test for 2 groups and when comparing three groups or more a Friedman’s test, with a Dunn’s multiple comparisons post-hoc test. The data are shown as median with interquartile range. The p values are demonstrated by * is p<0.05, ** is p<0.01 and **** is p<0.0001. (TIFF) [file pone.0256545.s009.tiff]

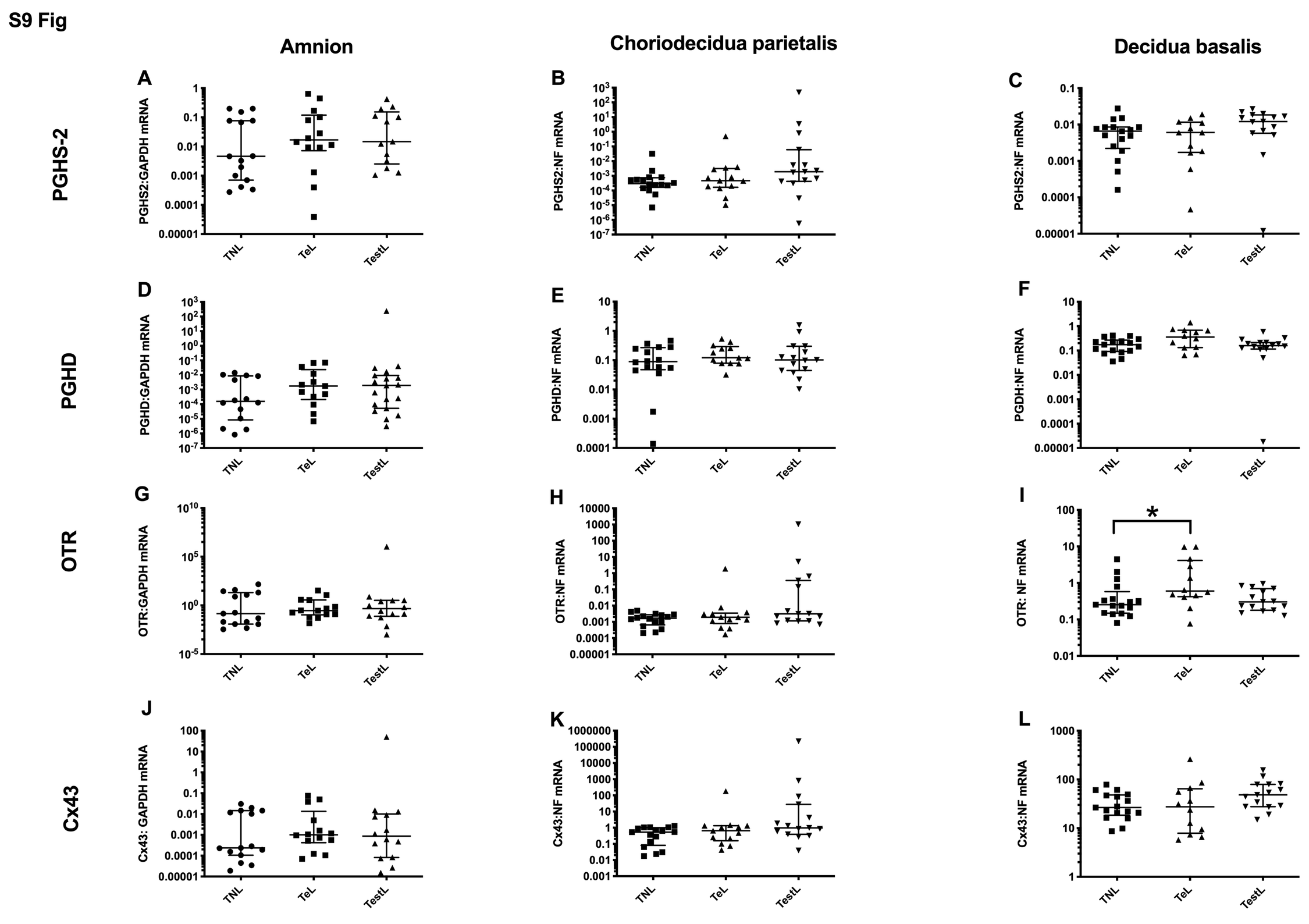

Supplement: S2 Fig — A subset of amnion, choriodecidual parietalis and decidua basalis samples were used for mRNA extraction. The samples were homogenised and RNA extracted and converted to cDNA. Copy numbers of PGHS-2, PGHD, OTR and CX43 mRNA for term no labour, term early and term established labour samples were measured quantitative rtPCR. Normally distributed data were analysed using an ANOVA followed by a Dunnett’s or Bonferroni’s post-hoc test for three groups or more. Data that were not normally distributed were analysed using a Friedman’s test, with a Dunn’s multiple comparisons post-hoc test. The data are shown as median with interquartile range. The p values are demonstrated by * is p<0.05 and ** is p<0.01. (TIFF) [file pone.0256545.s010.tiff]

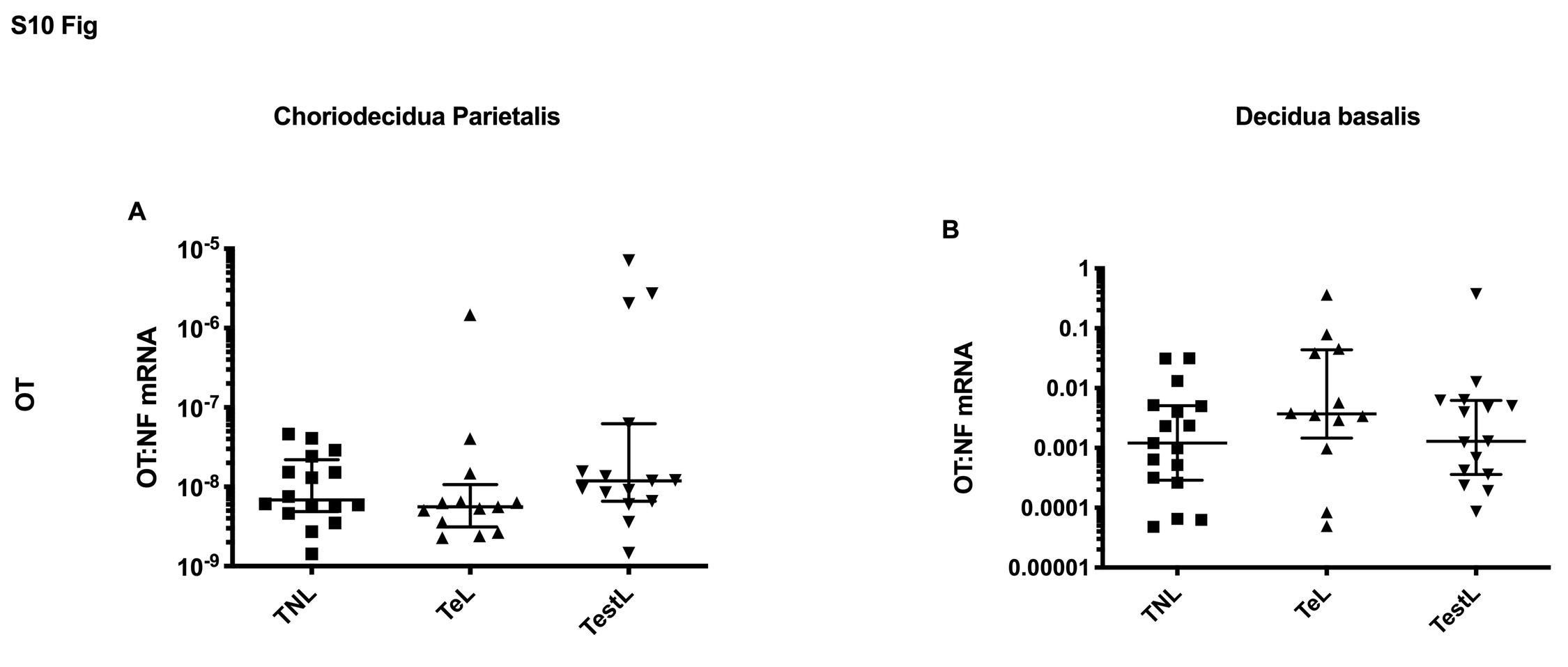

Supplement: S3 Fig — A subset of, choriodecidua parietalis and decidua basalis samples were used for mRNA extraction. Choriodecidua parietalis: term no labour (TNL; 39.3 ± 0.9 weeks, n = 16), early labour (38.3 ± 1.1 weeks, n = 13) and term established labour (39.5 ± 1.0, n = 15). Decidua basalis: term no labour (TNL; 39.3 ± 0.9 weeks, n = 17), early labour (38.7 ± 1.3 weeks, n = 12) and term established labour (39.5 ± 1.0, n = 15). The samples were homogenised and RNA extracted and converted to cDNA. Copy numbers of OT mRNA for the term no labour, term early and term established labour samples were measured quantitative rtPCR. Normally distributed data were analysed using an ANOVA followed by a Dunnett’s or Bonferroni’s post-hoc test for three groups or more. Data that were not normally distributed were analysed using a Friedman’s test, with a Dunn’s multiple comparisons post-hoc test. The data are shown as median with interquartile range. The p values are demonstrated by * is p<0.05 and ** is p<0.01. (TIFF) [file pone.0256545.s011.tiff]
